# A quantitative report on the impact of chloride on the kinetic coefficients of auxin-induced growth: a numerical contribution to the “acid growth hypothesis”

**DOI:** 10.1186/s40064-016-3626-y

**Published:** 2016-11-15

**Authors:** Mariusz Pietruszka, Aleksandra Haduch-Sendecka

**Affiliations:** Plant Physiology, Faculty of Biology and Environment Protection, University of Silesia, Katowice, Poland

**Keywords:** Auxin, Coleoptile, Elongation growth, Growth rate, Maize, Membrane potential, pH, Protons

## Abstract

This work presents the application of several our own novel methods of analysing the kinetics of plant growth, which create, among others, a common platform for the comparison of experimental results. A relatively simple formula is used to parameterize the wide range of data that has been obtained for *Zea mays* L. in the literature, though it can also be used for different species. A biophysical/biochemical interpretation of the parameters was obtained from a theoretical model that is based on a modified Lockhart equation. The derived formula, which was extended for practical use in Zajdel et al. (Acta Physiol Plant 38:5, 2016), and which was implemented in the attached computer program (ibid.), allowed the data that was obtained from the growth-related problems to be parameterized in a simple way. As a working example that shows the robustness of our approach, we comment in detail on the qualitative assessments of the impact of chloride ions on auxin-induced growth. We note that calculated continuous curves (fits), which are rooted in the growth functional that was introduced by Pietruszka (J Theor Biol 315:119–127, 2012), were in a perfect agreement (R^2^ ~ 0.99998) with the raw experimental data that was published recently by Burdach et al. (Ann Bot 114:1023–1034, 2014). This fact justified the use of this strict technique, which allows for the determination of kinetic coefficients, to critically evaluate the results and suppositions (claims) therein. Moreover, we calculated the time-delay derivative of elongation growth—pH cross-correlations, and validated the “acid growth hypothesis” in figures by considering, amongst others, the magnitude of the H^+^-activity of elongation growth (per μm). An empirical constant (field strength), E_H+_ = E_m_/(log_10_ 1/a_H+_ ∙ μm) = 0.157 ± 0.009 [V/mm] was obtained, where E_m_ [mV] is the membrane potential in the perenchymal coleoptile cells of *Zea mays* L. When this relation is known, the membrane potential can not only be determined for intact growth, but also for different intervening substances exclusively from growth (or growth rate) and pH measurements, i.e. *without* performing electrophysiological measurements. However, the question of whether this constant is universal remains open.

**Electronic supplementary material:**

The online version of this article (doi:10.1186/s40064-016-3626-y) contains supplementary material, which is available to authorized users.

## Background

The expansion dynamics of plant cells and organs, especially the coleoptiles of maize (*Zea mays* L.) as a model system, have been a hot topic of debate for many decades (Kutschera and Schopfer 1985a, b), particularly in the context of the independent action of auxin (indole-3-acetic acid, IAA) that was proposed by Cleland (1971) and Hager et al. (1971) in the form of the “hypothesis of acid growth”. Hager’s wall acidification model is based on experiments using the shoots of grass seedlings (coleoptiles, which are leaf-like axial organs). Since then, the hypothesis has been carefully evaluated by many scientists (e.g., Hager 2003; Kutschera 1994, 2003; Lüthen et al. 1990; Lüthen and Böttger 1993).

The theory that the naturally occurring plant hormone auxin (IAA) may initiate coleoptile elongation by rapidly lowering the apoplastic pH value, which is known as “acid growth hypothesis”, was based on the following observations (Kutschera 2006): (1) acidic buffers (pH 3.5–4.0) elicit a rapid short-term growth response of coleoptiles (2) IAA enhances the rate of proton extrusion so that pH of about 5.0 is established in the walls and (3) metabolic inhibitors block both hormone-mediated wall acidification and cell elongation. However, it was advocated by Kutschera (1994, 2006) that the fungal phytotoxin fusicoccin (FC) not IAA fulfills the pre-conditions of this theory. This controversy has continued to this day in the form of an ongoing debate (Kutschera 2006), even though evidence has accumulated that the final target of auxin action is the plasma membrane H^+^-ATPase, which excretes H^+^ ions into the cell wall compartment and takes up K^+^ ions in the antiport through an inwardly rectifying K^+^ channel (Hager 2003; see also Steinacher et al. 2012 for auxin dynamics). The pumping of auxin-amplified H^+^ decreases the cell wall pH, activates pH-sensitive enzymes and proteins in the wall, and initiates cell-wall loosening, wall-creep and extension growth. These processes can be blocked by a voltage inhibition of H^+^-ATPase by neutralizing K^+^ ions.

The acid growth hypothesis states that the H^+^ ions that are excreted into the apoplast act as wall-loosening factors (WLF) via the activation of hydrolytic enzymes. This mechanism, which involves enzymes in cell-wall-loosening process, may occur via the hydrolysis of covalent bonds or the disruption of non-covalent bonds. Following Hager (2003), examples of pH-dependent yielding mechanisms of the cell wall include: (*i*) expansins (Cosgrove 1993), (*ii*) xyloglucans (Fry et al. 1992) and (*iii*) yieldins (Okamoto-Nakazato et al. 2001), all of which are activated by acid conditions.

Expansins do not fit the idea of a wall loosening enzyme as they have no effect on wall extension at neutral pH; they achieve a high activity at pH 3.5–4.5. Expansins appear to increase polymer mobility in the cell wall by breaking wall hydrogen bonds, thereby allowing the microfibrils to slip into the wall matrix throughout extension (McQueen-Mason and Cosgrove 1994; Cosgrove 2000). Expansin loosens the network-like connections between the cellulose microfibrils within the cell wall, which allows the cell volume to increase via turgor and osmosis. A typical sequence leading up to this would involve the introduction of a plant hormone that causes protons (H^+^ ions) to be pumped out of the cell into the cell wall. As a result, the cell wall solution becomes more acidic. This activates expansin activity, thus causing the wall to become more extensible and to undergo wall stress relaxation, which enables the cell to take up water and to expand (Rayle and Cleland 1992; Yennawar et al. 2006).

Xyloglucans are quantitatively important hemicelluloses within the cell wall and can be incorporated into and bound to the surface of cellulose microfibrils (Hager 2003). Xyloglucans play an important role in the control of cell growth because they probably influence cell wall extensibility and, therefore, the rate of cell expansion during cell wall loosening. Moreover, xyloglucans can be broken down into a fucose-containing oligosaccharide which exerts a hormone-like anti-auxin effect on growth (Fry 1989).

Yieldins, which are wall-bound proteins, are involved in growth regulation (Okamoto and Okamoto 1994; Okamoto-Nakazato et al. 2000a, b). It is interesting that in the course of cell wall loosening (called yielding), the yield threshold *Y* is the critical tension beyond which irreversible extension begins, the energy (corresponding to *Y*) to split bonds between microfibrils can be lowered by the protein yieldin. This fact is also reflected in acidic pH (see also Eq. (18) in Pietruszka 2012, where the functional dependence of *Y* = *Y* [*n*] is introduced). Summarizing, auxin, FC or acid buffers have a similar impact on the cell-wall-loosening processes (Pietruszka and Haduch-Sendecka 2016).

**Fig. 1:**
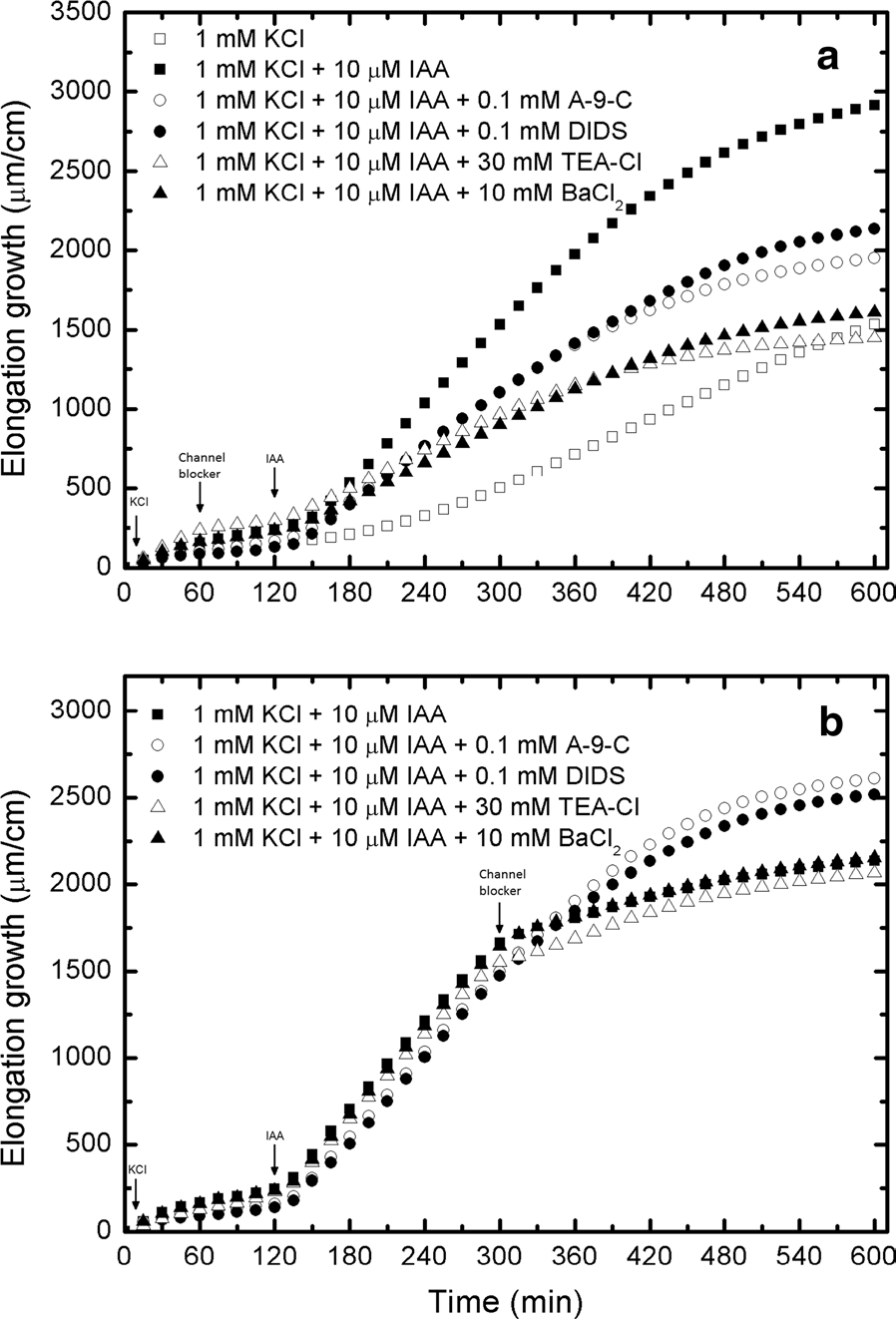
**a** Total elongation growth for growth rate measurements as shown in Fig. 2 (Burdach et al. 2014), and calculated numerically as a cumulative integral Eq. (6). **b** Total elongation growth for growth rate measurements as shown in Fig. 3 (Burdach et al. 2014), and calculated numerically as a cumulative integral Eq. (6)

Several models have recently been proposed to portray this interdependency in mathematical terms. We only mention a few of them here. A refined hormone model of primary root growth where the wall extensibility is determined by the concentration of a wall enzyme, whose production and degradation are assumed to be controlled by auxin and cytokinin, was proposed by Chavarria-Krauser et al. (2005). More recently, Pietruszka (2012) formulated a biosynthesis/inactivation model for enzymatic WLFs (Wall Loosening Factors) or non-enzymatically mediated cell evolution that is based on the Lockhart/Ortega formalism, where the physiology and biochemistry of the growth process were related by analytical equations that allowed very high fidelity factors (determination coefficient *R* ^2^ ≈ 0.99998, regression *P* < 0.0001) with the empirical data to be acquired. Also, in the same context of biosynthesis, biological growth as a resultant effect of three forms of energy (mechanical, thermal and chemical) and their individual couplings was summarized in the form of a theoretical framework by Barbacci et al. (2013). In this description biological growth was the effect of three forms of energy and their couplings (noted as M/T, M/C and T/C with M for Mechanical, T for Thermal and C for Chemical). Each couple of intensive and extensive variables for each energy was linked by one component of Tisza’s matrix (defined in Eq. 5, ibid.) and further extensions of the model. A proposed function of each form of energy and coupling was provided (Fig. 1, ibid.). The derivation, though elegant and sophisticated, requires many parameters and externally controlled turgor pressure *P* and temperature *T* in order to retrieve the data that is extracted from Proseus and Boyer (2008) experiment numerically (see Fig. 5 in Barbacci et al. 2013).

Quite recently, a novel effective formula for the parameterization of the growth kinetics of plants was derived from the modified Lockhart/Ortega type of equation (Zajdel et al. 2016). The formula allows for the greater transferability and quantitative comparability of experimental results. Its applicability has been successfully tested on literature data for *Arabidopsis thaliana* and *Zea mays* L. (ibid.). The analysis allows, among others, the values of the diffusion rate *k*_2_ to be obtained. A comparison of *k*_2_ values that were obtained from the fits was carried out, which quantified the trends that are caused by different experimental conditions. An easy to use free computer program (Zajdel et al. 2016) has been written that demonstrates the use of the function and to aid in the application of the formula in community of plant physiologists.

At the end, we must stress that investigating the “acid growth hypothesis” and resolving the mounting controversies is still impossible today using solely biological experiments. It can only be explored further using in silico methods as shown in this work. In this case the “experimentum crucis” for the biological problem (acid growth hypothesis) paradoxically may belong to physics and mathematics.

In the next Section we present all of the methods that we used for the investigation of growth data.

## Methods

### Calculations of kinetic coefficients

For an indispensable introduction to the major method that was used in this work, we followed Zajdel et al. (2016), though the narrative is based on an earlier notion (Pietruszka 2012, see Appendix) that the expression for the growth factor concentration can be derived from the sum of the growth factor biosynthesis (production rate) *k*_1_ and an inactivation-like (diffusive) part with a proportionally constant *k*_2_:

1 $$\frac{dn(t)}{dt} = k_{1} - k_{2} n(t)$$

One has to note that analogous models have already been introduced—Rojas et al. (2011) used similar terms to describe the kinetics of pectin chemistry in the oscillatory growth of pollen tubes. In this context, *k*_1_ would be the equivalent of the de-esterification and *k*_2_ would incorporate cross-linking, dilution by deposition and advection terms. We did not want to limit our approach to any specific factors such as calcium pectates (Proseus and Boyer 2006) or hormones (Chavarria-Krauser et al. 2005). Therefore, the formula that was presented as Eq. (21) in Pietruszka (2012) and broadly employed in this work is of general applicability. Note, that combining *n*_0_ and *k*_1_ naturally follows from the initial form of the equation, where dimensional consistency requires [*k*_1_] = concentration∙time^−1^ and [*k*_2_] = time^−1^.

In our recent paper (Zajdel et al. 2016), we showed that Eq. (21) in Pietruszka (2012) can be utilized to adequately predict the shape of the relative growth function for time scales that are comparable to those of cell wall biosynthesis. It can also describe the primary “lag” region, which is approximately the linear growth of the cell due to initially active factors.

We further recall that the formula given by Eq. (21), ibid., can be simplified by assuming time constants *T*_1_ ≫ *T*_2_ > 0 (which are the reciprocals to *k*_1_ and *k*_2_, respectively), which is equivalent to the statement that the biosynthesis rate *k*_1_ is much smaller than the reduction rate (e.g., transport into the cell wall) *k*_2_.

Previously (Zajdel et al. 2016), we also proposed dividing the time scale (of the living cell or organ) into two epochs.

(*i*) when only biosynthesis mechanisms are initially the limiting factors (*t*/*T*_2_ is negligible), the solution yields:

2 $$\frac{{V(t) - V_{0} }}{{V_{0} }} = e^{{\overline{\Phi }_{0} \left( {t + \frac{1}{2}\frac{{t^{2} }}{{{\rm T}_{1} }}} \right)}} - 1 = e^{{\overline{\Phi }_{0} t\left( {1 + \frac{1}{2}\frac{t}{{{\rm T}_{1} }}} \right)}} - 1 \approx \overline{\Phi }_{0} t + \overline{\Phi }_{0} \frac{1}{2}\frac{{t^{2} }}{{{\rm T}_{1} }} = At + B_{1}$$

where $\overline{\Phi }_{0} = \Phi_{0} (P - Y)n_{0} ,$ and *Φ*_0_ is a Lockhart constant.

(*ii*) when the diffusion mechanisms are dominant:

3 $$\frac{{V(t) - V_{0} }}{{V_{0} }} = e^{{\overline{\Phi }_{0} \frac{{{\rm T}_{2} }}{{{\rm T}_{1} }}\left( {{\rm T}_{1} - {\rm T}_{2} } \right)}} e^{{^{{\overline{\Phi }_{0} \frac{{{\rm T}_{2} }}{{{\rm T}_{1} }}\left( {{\rm T}_{1} - {\rm T}_{2} } \right)}} e^{{\frac{ - t}{{{\rm T}_{2} }}}} }} e^{{^{{\overline{\Phi }_{0} \frac{{{\rm T}_{2} }}{{{\rm T}_{1} }}t}} }} - 1$$

and utilizing *T*_1_ ≫ *T*_2_ > 0 (*T*_1_ − *T*_2_ ≈ *T*_1_) as well as assuming that *t*/*T*_1_ still remains small, we get the dominant term:

4 $$\frac{{V(t) - V_{0} }}{{V_{0} }} \approx const^{*}e^{{\overline{\Phi }_{0} \frac{{{\rm T}_{2} }}{{{\rm T}_{1} }}\left( {{\rm T}_{1} - {\rm T}_{2} } \right)}} e^{{^{{ - \overline{\Phi }_{0} \frac{{{\rm T}_{2} }}{{{\rm T}_{1} }}\left( {{\rm T}_{1} - {\rm T}_{2} } \right)e^{{\frac{ - t}{{{\rm T}_{2} }}}} }} }} - 1 \approx Ce^{{ - Fe^{ - Dt} }} + B_{2} = Ce^{{ - e^{{ - D(t - t_{e} )}} }} + B_{2}$$

As a last step, we neglected the correlations between factors (*i*) and (*ii*) and added both contributions, thereby arriving at the semi-empirical formula that is derived from an approximated solution of the modified Ortega (1985) equation, though ultimately rooted in Eq. (1):

5 $$\frac{{V(t) - V_{0} }}{{V_{0} }} = At + B + Ce^{{ - e^{{ - D\left( {t - t_{e} } \right)}} }}$$

Equation (5) describes the relative growth curves that are prompted by external biotic and abiotic stimuli and is the sum of the linear ‘start-up’ region and nonlinear accelerated growth. Here, we introduced a *t*_e_ parameter (an effective time) in all of the cases in which a growth factor is added to the plant incubation medium. This characteristic time (*t*_e_) cannot be directly equated with the time of the addition of an exogenous factor but rather as the effective moment at which it starts to dominate. Fitting equation Eq. (5) to the experimental data provides the initial parameters *A*, *B*, *C*, *D* and *t*_e_, under the condition that the proper unit scaling was done (Zajdel et al. 2016). It seems that parameter *C* = *C*(*T*), if dependent on temperature, is also biologically meaningful. It can be successfully described by the Euler beta distribution or (equivalently) by pH changes in the apoplast as a result of proton (H^+^ ions) extrusion into the cell wall compartment (Pietruszka 2016).

We already identified parameter *D* as 1/*T*_2_ and obtained the first estimate of $\overline{\Phi }_{0}$ from parameter *A*. At this approximation level, parameter *C* can only be used to quantify growth as an equivalent of ‘growth amplitude’; *C* can be roughly associated with *k*_2_ (*T*_2_) through *C* ~ exp(*T*_2_) ~ exp(1/*k*_2_) but this would be valid only in the epoch in which a diffusion mechanism is dominant (nonlinear). Parameter *B* includes all of the constants and the slowly varying orders of expansion and is of no theoretical use. The proportionality factor *n*_0_ comes from the initial concentration the *n*_0_ of the growth factors at *t* = 0. Within the current approximation, *A* ~ Φ_0_(*P*–*Y*)*n*_0_, and *A* should be linearly dependent on the concentration *n*_0_ and parameter *D* to *k*_2_ (s^−1^). Note, that the fitting parameters have the proper dimensions and require the *y*-axis to be a dimensionless relative elongation and the *x*-axis should denote the time in seconds. For further elucidation concerning the details of the method we suggest here, see Zajdel et al. (2016).

We note that in some of the cases that are considered in this work, the relevant interacting coupling strengths (*k*) must be taken into account when extending the model. The latter must be considered for different, but not mutually interacting growth factors (e.g., plant hormones function independently of other hormones), which should be reflected in the structure of the growth rate spectrum. In the extended form, the Ortega equation for the relative (logarithmic) volumetric growth for several (*n*) growth factors (Pietruszka 2012) reads

6 $$\frac{1}{V(t)}\frac{dV(t)}{dt} = \Phi_{0} \sum\limits_{i = 1}^{n} {\frac{1}{{w_{i} }}x_{i} (t)} (P(t) - Y[n_{i} (t)]) + \frac{1}{\varepsilon }\frac{dP(t)}{dt}$$

where the completeness relation ∑ _*i*=1_^*n*^ *w*_*i*_ = 1 imposes a constraint for the positive weights *w*_i_ of the *i*th constituent (wall loosening factor or WLF for short, which may be of an either endogenous or exogenous origin). Here *x*_i_ denotes the *i*th constituent concentration. In Eq. (6) the Lockhart (1965) constant Φ_0_ is responsible for cell wall viscoplastic extensibility, while ε is the elastic constant (Ortega 1985). Note, the second order correction for the yield stress *Y* enters Eq. (6) via functional dependence. As it was already noted (ibid.), the main strength of the model Eq. (6) is that it handles in vivo and in vitro behaviour in similar terms.

### Spectral density calculations

Recalling Eq. (6) for multiple growth factors (such as the anion A-9-C or DIDS and cation channel blockers TEA-Cl or BaCl_2_ that were introduced together with IAA), we may rewrite it in first approximation by neglecting the elastic term. We only assume the viscoplastic extension at a constant turgor pressure *P*–*Y* > 0. The turgor threshold is presumed to be constant, though it may change slightly (Schopfer 2006; Pietruszka 2012). Hence, we obtain an explicit formula, which can be utilized for A-9-C and IAA, as an example

7 $$\frac{1}{V(t)}\frac{dV(t)}{dt} = \Phi_{0} \left( {\left( {\frac{1}{{w_{A - 9 - C} }}x_{A - 9 - C} e^{{ - k_{A - 9 - C} t}} + \frac{1}{{w_{IAA} }}x_{IAA} e^{{ - k_{IAA} t}} } \right)(P(t) - Y)} \right)$$

**Fig. 2:**
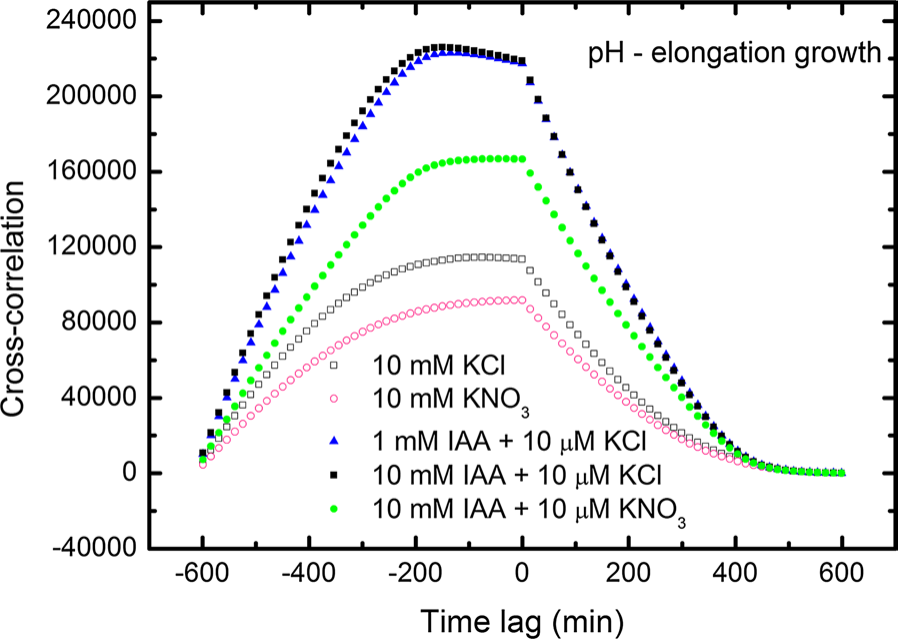
Cross-correlations of pH and elongation growth as a function of time lag τ (min). Analysed data for the insets in Fig. 4 (Burdach et al. 2014)

The solution is *not* exponentially divergent, unlike to the Lockhart (1965) equation, but belongs to the class of double exponent functions. The additive part in parenthesis transforms into the multiplication of the exponents (e^A+B^ = e^A^e^B^) that preserve the double exponent, a sigmoid-like form of the solution Eq. (5). This logarithmic differential equation for the extending volume (here, of the coleoptile segment) can be written under the assumption that the channel blockers alone do not interact with each other. (Similar equations can be obtained for the remaining combinations of IAA with anion or cation blockers). By recalling the Fourier decomposition method that was adopted specifically in Haduch-Sendecka et al. (2014) and Pietruszka and Haduch-Sendecka (2016), we can denote the left side of Eq. (7) simply by GR (volumetric Growth Rate) and calculate the Fourier transform for both sides of this equation. The contributions of individual components can be split into their respective Fourier transforms (Harris 1998)

8 $$\begin{aligned} F(GR) \propto \frac{1}{{k_{A - 9 - C} + 2\pi if}} \hfill \\ F(GR) \propto \frac{1}{{k_{IAA} + 2\pi if}} \hfill \\ \end{aligned}$$

(where *i* = √−1 and *f* stands for the frequency) while the input into the energy distribution spectrum is proportional to

9 $$\begin{aligned} P_{A - 9 - C} (GR) \propto \frac{1}{{k_{A - 9 - C}^{2} + 4\pi^{2} f^{2} }} \hfill \\ P_{IAA} (GR) \propto \frac{1}{{k_{IAA}^{2} + 4\pi^{2} f^{2} }} \hfill \\ \end{aligned}$$

where *P* stands for the power spectral density (reflecting process intensity—not to be confused with turgor pressure *P*). The latter two components, which are viscoplastic in origin, will be maximum at *f* = 0 Hz and have a Lorentz form for higher frequencies. It is very unlikely that they will contribute to the oscillations and the central part should be subtracted by detrending prior to the Fourier procedure. It is worth noting that any appearance of growth oscillations (*f* > 0) will compete for the spectral density that is allocated to this part. The results for *f* = 0 can be read off from Additional file 1: SI Figs. 9A and B, which correspond to Figs. 2 and 3 in Burdach et al. (2014), and are collected in Fig. 9a, b. Note, that the power spectrum at *f* = 0 is proportional to 1/*k* ^2^, thus allowing for an estimation of the process intensity for different treatments.

### Evaluation of turgor pressure

As was already mentioned, *A* ~ Φ_0_(*P* − *Y*)*n*_0_ in Eq. (5) and *A* should be linearly dependent on the concentration *n*_0_. The proportionality factor *n*_0_ comes from the initial concentration *n*_0_ of the growth factors at *t* = 0. Because Φ_0_ is a Lockhart constant that is equal 10^−6^ [1/MPa∙s] and the turgor threshold *Y* is assumed to be constant throughout, *A*—coefficient is simply proportional to the turgor pressure *A* ~ *P* [MPa], see Tables 1, 2, 3, 4, 5, 6, 7, 8, 9 and 10. Note, when a WLF is introduced into a wall, it begins to act on some mechanically significant components to change either the steady state viscosity (~ 1/Φ) or the yield stress *Y* or both (Schopfer 2006). The relative volume, Eq. (5), increases (in fact it is effectively introduced into the system at *t*_e_) almost as fast as these parameters change.

**Table 1:** Fit parameters for the coleoptile of maize grown under constant dim green light at 25 °C

	*A* (s^−1^)	*B*	*C*	*D* = *k*_2_ (s^−1^)	*t*_e_ (s)
Polak et al. (2012): Fig. 2					
◆ Control	(14.0 ± 1.0)e−07	0.0017 ± 0.0006	0.087 ± 0.005	(12.6 ± 0.6) e−05	22,330 ± 160
Rudnicka et al. (2014): Fig. 1					
□ Control	(16.7 ± 0.7)e−07	0.0002 ± 0.0004	0.079 ± 0.003	(13.2 ± 0.5) e−05	22,080 ± 180
★ Mean control coefficients (calculated from the data above)	(15.4 ± 1.0)e−07	0.0009 ± 0.0006	0.083 ± 0.005	(12.9 ± 0.6) e−05	22,205 ± 180

**Table 2:** Fit parameters for the coleoptile of maize grown under constant dim green light at 25 °C and the influence of anion (A-9-C and DIDS) and cation (TEA-Cl and BaCl_2_) channel blockers that were implemented after 1 h, and then incubated in the presence of 10 μM IAA after 2 h

Burdach et al. (2014): Fig. 2	*A* (s^−1^)	*B*	*C*	*D* = *k*_2_ (s^−1^)	*t*_e_ (s)
□ 1 mM KCl	(13.8 ± 0.5)e−07	0.0042 ± 0.0002	0.132 ± 0.003	(10.3 ± 0.2)e−05	23,985 ± 95
■ 1 mM KCl + 10 μM IAA	(21.5 ± 2.1)e−07	0.0046 ± 0.0006	0.221 ± 0.008	(15.5 ± 0.4)e−05	15,640 ± 35
◯ 1 mM KCl + 10 μM IAA + 0.1 mM A-9-C	(12.1 ± 1.4)e−07	0.0033 ± 0.0004	0.155 ± 0.005	(15.9 ± 0.4)e−05	14,649 ± 32
● 1 mM KCl + 10 μM IAA + 0.1 mM DIDS	(0.0 ± 5.0)e−07	0.0029 ± 0.0007	0.230 ± 0.022	(12.1 ± 0.6)e−05	15,821 ± 65
△ 1 mM KCl + 10 μM IAA + 30 mM TEA-Cl	(0.0 ± 4.6)e−07	0.0098 ± 0.0013	0.145 ± 0.020	(12.1 ± 1.0)e−05	12,580 ± 150
▲ 1 mM KCl + 10 μM IAA + 10 mM BaCl_2_	(19.2 ± 1.9)e−07	0.0066 ± 0.0005	0.092 ± 0.007	(15.7 ± 0.8)e−05	14,881 ± 70

**Table 3:** Fit parameters for the coleoptile of maize grown under constant dim green light at 25 °C and the influence of anion (A-9-C and DIDS) and cation (TEA-Cl and BaCl_2_) channel blockers, and incubated in the presence of 10 μM IAA after 2 h, and then channel blockers implemented after 3 h

Burdach et al. (2014): Fig. 3	*A* (s^−1^)	*B*	*C*	*D* = *k*_2_ (s^−1^)	*t*_e_ (s)
■ 1 mM KCl + 10 μM IAA	(17.1 ± 0.8)e−07	0.0082 ± 0.0004	0.146 ± 0.002	(27.3 ± 0.5)e−05	11,580 ± 33
◯ 1 mM KCl + 10 μM IAA + 0.1 mM A-9-C	(0.0 ± 3.2)e−07	0.0062 ± 0.0006	0.268 ± 0.013	(14.5 ± 0.4)e−05	14,660 ± 40
● 1 mM KCl + 10 μM IAA + 0.1 mM DIDS	(7.0 ± 2.2)e−07	0.0031 ± 0.0005	0.232 ± 0.008	(15.8 ± 0.4)e−05	14,420 ± 33
△ 1 mM KCl + 10 μM IAA + 30 mM TEA-Cl	(20.7 ± 0.6)e−07	0.0038 ± 0.0003	0.131 ± 0.002	(27.3 ± 0.5)e−05	11,618 ± 28
▲ 1 mM KCl + 10 μM IAA + 10 mM BaCl_2_	(17.3 ± 0.8)e−07	0.0078 ± 0.0004	0.147 ± 0.002	(26.9 ± 0.6)e−05	11,837 ± 32

**Table 4:** Fit parameters for the coleoptile of maize grown under constant dim green light at 25 °C and the influence of 1 mM KCl or KNO_3_, and incubated in the presence or absence of 10 μM IAA

Burdach et al. (2014): Fig. 1A	*A* (s^−1^)	*B*	*C*	*D* = *k*_2_ (s^−1^)	*t*_e_ (s)
□ 1 mM KCl	(16.2 ± 1.3)e−07	0.0037 ± 0.0007	0.119 ± 0.009	(11.1 ± 0.7)e−05	24,100 ± 320
◯ 1 mM KNO_3_	(9.5 ± 1.5)e−07	0.0025 ± 0.0006	0.047 ± 0.011	(9.3 ± 1.6)e−05	24,690 ± 960
■ 1 mM KCl + 10 μM IAA	(22.7 ± 5.0)e−07	0.0030 ± 0.0015	0.219 ± 0.019	(15.6 ± 0.9)e−05	15,611 ± 88
● 1 mM KNO_3_ + 10 μM IAA	(28.7 ± 4.9)e−07	0.0005 ± 0.0013	0.092 ± 0.019	(14.7 ± 2.0)e−05	15,550 ± 190

**Table 5:** Fit parameters for the coleoptile of maize grown under constant dim green light at 25 °C and the influence of 10 mM KCl or KNO_3_, and incubated in the presence or absence of 10 μM IAA

Burdach et al. (2014): Fig. 1B	*A* (s^−1^)	*B*	*C*	*D* = *k*_2_ (s^−1^)	*t*_e_ (s)
□ 10 mM KCl	(17.1 ± 1.2)e−07	0.0022 ± 0.0006	0.066 ± 0.055	(13.5 ± 0.9)e−05	20,610 ± 200
◯ 10 mM KNO_3_	(18.0 ± 7.4)e−07	0.0013 ± 0.0007	0.033 ± 0.038	(9.4 ± 5.2)e−05	15,070 ± 750
■ 10 mM KCl + 10 μM IAA	(15.0 ± 11.0)e−07	0.0001 ± 0.0024	0.330 ± 0.043	(14.5 ± 1.2)e−05	15,070 ± 120
● 10 mM KNO_3_ + 10 μM IAA	(31.6 ± 3.2)e−07	−0.0002 ± 0.0011	0.085 ± 0.011	(19.9 ± 2.2)e−05	12,990 ± 180

**Table 6:** Fit parameters for the coleoptile of maize grown under constant dim green light at 25 °C and the influence of 10 mM KCl or 10 mM KNO_3_, and incubated in the presence or absence of 10 μM IAA

Burdach et al. (2014): Fig. 4	*A* (s^−1^)	*B*	*C*	*D* = *k*_2_ (s^−1^)	*t*_e_ (s)
□ 10 mM KCl	(12.7 ± 2.1)e−07	−0.0005 ± 0.0004	0.074 ± 0.011	(10.3 ± 0.8)e−05	18,850 ± 150
◯ 10 mM KNO_3_	(9.3 ± 1.4)e−07	0.0001 ± 0.0003	0.051 ± 0.006	(11.3 ± 0.8)e−05	17,950 ± 120
▲ 1 mM KCl + 10 μM IAA	(26.4 ± 0.9)e−07	−0.0050 ± 0.0006	0.084 ± 0.002	(40.5 ± 2.0)e−05	11,477 ± 72
■ 10 mM KCl + 10 μM IAA	(28.2 ± 1.3)e−07	−0.0057 ± 0.0008	0.090 ± 0.004	(34.1 ± 2.1)e−05	11,769 ± 95
● 10 mM KNO_3_ + 10 μM IAA	(21.4 ± 0.6)e−07	−0.0038 ± 0.0005	0.050 ± 0.002	(53.0 ± 3.7)e−05	11,050 ± 87

**Table 7:** Fit parameters for the coleoptile of maize grown under constant dim green light at 25 °C and the influence of 10 mM KCl, 10 mM KNO_3_ and 5 mM KCl plus 5 mM KNO_3_, and incubated in the presence or absence of 10 μM IAA

Burdach et al. (2014): Fig. 5	*A* (s^−1^)	*B*	*C*	*D* = *k*_2_ (s^−1^)	*t*_e_ (s)
■ 10 mM KCl + 10 μM IAA	(21.7 ± 1.8)e−07	0.0015 ± 0.0012	0.073 ± 0.005	(36.9 ± 4.2)e−05	11,450 ± 170
● 10 mM KNO_3_ + 10 μM IAA	(19.9 ± 1.3)e−07	0.0017 ± 0.0001	0.043 ± 0.003	(55.0 ± 11.0)e−05	10,780 ± 230
▲ 5 mM KCl + 10 μM IAA + 5 mM KNO_3_	(21.9 ± 2.3)e−07	0.0017 ± 0.0001	0.085 ± 0.006	(32.1 ± 3.6)e−05	11,740 ± 180

**Table 8:** Fit parameters for the coleoptile of maize grown under constant dim green light at 25 °C and the influence of anion (A-9-C or DIDS) and cation (TEA-Cl), and incubated in the presence of 10 μM IAA

Burdach et al. (2014): Fig. 6	*A* (s^−1^)	*B*	*C*	*D* = *k*_2_ (s^−1^)	*t*_e_ (s)
■ 10 mM KCl + 10 μM IAA	(28.0 ± 1.3)e−07	0.0001 ± 0.0007	0.090 ± 0.005	(33.5 ± 2.0)e−05	11,722 ± 92
◯ 10 mM KCl + 10 μM IAA + 0.1 mM A-9-C	(20.0 ± 0.5)e−07	−0.0021 ± 0.0004	0.056 ± 0.001	(45.2 ± 2.1)e−05	10,766 ± 64
● 10 mM KCl + 10 μM IAA + 0.1 mM DIDS	(17.7 ± 0.4)e−07	−0.0022 ± 0.0004	0.045 ± 0.001	(47.2 ± 2.6)e−05	10,000 ± 77
△ 10 mM KCl + 10 μM IAA + 30 mM TEA-Cl	(14.2 ± 0.5)e−07	−0.0021 ± 0.0004	0.044 ± 0.001	(45.0 ± 2.9)e−05	11,219 ± 88

**Table 9:** Fit parameters for the coleoptile of maize grown under constant dim green light at 25 °C and the influence of A-9-C and TEA-Cl added together or separately (1 h after starting the experiment), and incubated in the presence of 10 μM IAA

Burdach et al. (2014): Fig. 7	*A* (s^−1^)	*B*	*C*	*D* = *k*_2_ (s^−1^)	*t*_e_ (s)
■ 10 mM KCl + 10 μM IAA	(28.3 ± 1.2)e−07	−0.0047 ± 0.0007	0.088 ± 0.003	(34.2 ± 2.0)e−05	11,802 ± 91
○ 10 mM KCl + 10 μM IAA + 0.1 mM A-9-C	(19.6 ± 0.5)e−07	−0.0016 ± 0.0004	0.057 ± 0.001	(46.0 ± 2.3)e−05	10,948 ± 67
▵10 mM KCl + 10 μM IAA + 30 mM TEA-Cl	(14.3 ± 0.5)e−07	−0.0012 ± 0.0004	0.043 ± 0.001	(46.3 ± 3.1)e−05	11,325 ± 90
●10 mM KCl + 10 μM IAA + 0.1 mM A-9-C + 30 mM TEA-Cl	(7.4 ± 0.9)e−07	0.0033 ± 0.0005	0.053 ± 0.003	(29.2 ± 1.9)e−05	12,540 ± 110

**Table 10:** Fit parameters for the coleoptile of maize grown under constant dim green light at 25 °C and the influence of an anion channel blocker (A-9-C) and 10 mM KCl or 10 mM KNO_3_ and 10 μM IAA, added to the medium at 2 h

Burdach et al. (2014): Fig. 8	*A* (s^−1^)	*B*	*C*	*D* = *k*_2_ (s^−1^)	*t*_e_ (s)
■ 10 mM KCl + 10 μM IAA	(28.0 ± 1.3)e−07	−0.0048 ± 0.0008	0.090 ± 0.004	(33.6 ± 2.0)e−05	11,683 ± 94
● 10 mM KNO_3_ + 10 μM IAA	(21.1 ± 0.6)e−07	−0.0029 ± 0.0005	0.050 ± 0.002	(51.4 ± 3.5)e−05	10,989 ± 85
□10 mM KCl + 10 μM IAA + 0.1 mM A-9-C	(20.2 ± 0.5)e−07	−0.0017 ± 0.0004	0.055 ± 0.001	(45.4 ± 2.1)e−05	10,697 ± 64
◯ 10 mM KNO_3_ + 10 μM IAA + 0.1 mM A-9-C	(14.5 ± 0.5)e−07	0.0005 ± 0.0004	0.052 ± 0.002	(43.5 ± 2.6)e−05	9865 ± 83

### Cross-correlations

In signal processing, cross-correlation is the measure of the similarity of two waveforms as a function of a time-lag that is applied to one of them. This is also known as a sliding dot product or sliding inner-product. For the continuous functions *f* and *g*, the cross-correlation is defined as:

10 $$(f * g)(\tau ) \equiv \int\limits_{ - \infty }^{\infty } {f^{*} (t)g(t + \tau )dt}$$

where f* denotes the complex conjugate of *f* and *τ* is the time lag. Note, that the cross-correlation is maximum at a lag equal to the time delay. Similarly, the cross-correlation for discrete functions is defined as:

11 $$(f * g)[n] \equiv \sum\limits_{m = - \infty }^{\infty } {f^{*} [m]g[m + n]}$$

the definition of which is utilized in this work (here: f* = *f*).

However, we may proceed even further and consider the first derivative of Eq. (10) with respect to the time shift τ. As a result we receive the following magnitude, where the prime denotes the time derivative, as usual:

12 $$\left( {f*g} \right)'\left( \tau \right) = \frac{d}{d\tau }(f * g)(\tau ) \equiv \frac{d}{d\tau }\left[ {\int\limits_{ - \infty }^{\infty } {f^{*} (t)g(t + \tau )dt} } \right]$$

By assuming *f* ≡ pH(*t*) (pH is the negative logarithm of the activity of the (solvated) hydronium ion or pH = − log_10_[H^+^] = log_10_[1/H^+^], which is sometimes expressed as the measure of the hydronium ion concentration), and *g* *≡* *u*(*t*) [μm]—elongation growth, we may calculate the convolution of these quantities

13 $$\left( {f*g} \right)'\left( \tau \right) = \frac{d}{d\tau }(f * g)(\tau ) \equiv - \frac{d}{d\tau }\left[ {\int\limits_{ - \infty }^{\infty } {\log_{10} [H^{ + } (t)]u(t + \tau )dt} } \right]$$

By assuming *f* ≡ pH(*t*), where pH is treated as a non-separable variable name and *g* ≡ *u*(*t*) [μm], the cross-correlation derivative (over time delay τ) can be calculated explicitly as follows

14 $$\frac{d}{d\tau }({\text{pH}} * u)(\tau ) \equiv \frac{d}{d\tau }\left[ {\int\limits_{ - \infty }^{\infty } {{\text{pH}}(t)u(t + \tau )dt} } \right] = \int\limits_{ - \infty }^{\infty } {{\text{pH}}(t)\underbrace {{\frac{d}{d\tau }u(t + \tau )}}_{u'}dt} = \int\limits_{ - \infty }^{\infty } {{\text{pH}}(t)u'(t + \tau )dt}$$

where *u*′ = *v* = d*u*/dτ.

A dimensional analysis of the first derivative, Eq. (12), has the following meaning—it designates the activity of H^+^ ions per micrometer, and presumably can be directly connected with the H^+^-efflux per μm. Hence, any jump at time delay *τ* = 0 will carry quantitative information about this magnitude. Obviously, the jump would be different with the application of diverse treatments, especially—cation and anion blockers, as was considered in Burdach et al. (2014) and reconsidered in this work. This is indeed the case.

The investigation was based on the data that was published by Burdach et al. (2014), and reconsidered by us for further examination.

### Supplemented data

Before we start quantitative analysis, we first need to supplement some of the raw experimental results Burdach et al. (2014).

**Fig. 3:**
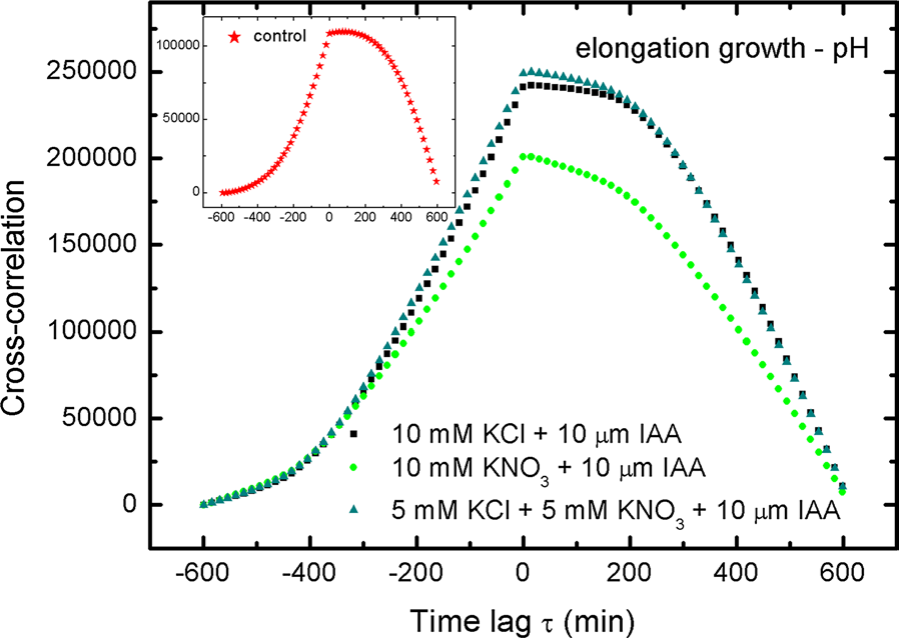
Cross-correlations of pH and elongation growth as a function of time lag τ (min). Analysed data for the insets in Fig. 5 (Burdach et al. 2014)

1. From the digitalization of figures published earlier (Polak et al. 2012: Fig. 2 and Rudnicka et al. 2014: Fig. 1), we introduced (Table 1; Additional file 1: SI Fig. 1) the appropriate control data, which should be the basis of any properly conducted data examination but which was absent in Burdach et al. (2014).
2. Since we have—*ν* = *u*′ (growth rate *ν* equals growth time derivative *u*′), we can calculate a cumulative integral numerically
  15 $$\int {vdt = \int {u^{{\prime }} dt = \int {\frac{du}{dt}dt = \int {du = u} } } }$$
  to retrieve the lacking (not presented by the authors) elongation growth curves in Figs. 2 and 3, which are otherwise the main basis in their examination (they focused their attention on these two figures). The results of our calculations (Eq. 7) are presented in Fig. 1a, b and Additional file 1: SI Figs. 2A, B.

### Accuracy of the results

We recall that fitting parameter *A*, which is numerically equal to *A* ~ Φ_0_(*P*−*Y*)*n*_0_, where *A* should be linearly dependent on concentration *n*_0_; the diffusion parameter *D* ~ *k*_2_ (s^−1^). Note, that both of these parameters, which are important for our analysis, acquire the least variance. Most importantly, the most vital parameter, *k*_2_ = *D*, has the relatively smallest error (see Tables 1, 2, 3, 4, 5, 6, 7, 8, 9 and 10; Additional file 1: SI Figs. 1–8), and therefore attains a high reliability.

Errors, as presented in the Tables, are calculated by standard deviation and total derivative methods.

## Results and discussion

### Control data

The results are presented in the charts and tables both in the main text and Supporting Information (a Navigation Additional file 1: SI Table 3 is supplied to aid the reader). The data from Table 1 show the coefficients *A*–*D* that were obtained for the growth of maize coleoptile segments in the APW (our control). These experiments were conducted under the same conditions and used the same equipment as did Burdach et al. (2014). It seems from the published data that Burdach’s group used IAA to accelerate growth and shorten the duration of the experiments. Moreover, the reason for using KCl (or KNO_3_) is to ensure an adequate concentration of ions (accessibility of K^+^ and Cl^−^ ions) to the coleoptile segments, the transport of which could have influenced the appropriate channel blockers. However, there is no evidence that IAA had any other impact than to speed up the response of maize growth in these studies. The use of several treatments in a single experiment obscures and makes drawing the correct conclusions considerably more difficult, and therefore it was necessary to use another (appropriate) control in order to subtract the background.

### Coefficients *A-D*

The ongoing analysis is based on the above-described methods.

Figure 1a, b were used to create fits to the data as shown in Additional file 1: SI Figs. 2A and SI 2B from Figs. 2 and 3 in Burdach et al. (2014), respectively. Thus obtained coefficients *A*–*D* are collected in Tables 2 and 3 for comparison.

Figures 1–8 from Burdach et al. (2014) were digitized using a GetData Graph Digitizer and then re-analysed using our method (fitexex) to obtain coefficients *A*–*D* (Tables 2, 3, 4, 5, 6, 7, 8, 9 and 10; Additional file 1: SI Figs. 3–8). For Figs. 2 and 3 (Burdach et al. 2014), we had to make an additional calculation in order to obtain data that was lacking for the elongation growth (see Fig. 1a, b and Additional file 1: SI Fig. 2A, B).

The authors state (Fig. 2 in Burdach et al. 2014) that the addition of chloride channel blockers (A-9-C and DIDS) diminished the elongation growth of maize the coleoptile segments by 32 and 25%, respectively. One can deduce from Table 2C that the influence of DIDS is similar to their control (1 mM KCl + 10 μm IAA), but also that BaCl_2_ is close to the control in the APW. The magnitude coefficient *C* for the other (TEA-Cl and A-9-C) channel blockers are more or less half distance between the both of the mentioned controls. Interestingly, the diffusion rate (*k*_2_) divides the data into two groups that are located around the two controls (the first group—their control, A-9-C, BaCl_2_ and the second group—the control in the APW, DIDS, TEA-Cl).

The authors (Fig. 3 in Burdach et al. 2014) also state that the addition of potassium channel blockers (TEA-Cl and BaCl_2_) reduced the elongation growth of the maize coleoptile segments by more than 50%. One can deduce from our Table 3C that the influence of BaCl_2_ is similar to their control (1 mM KCl + 10 μm IAA), while TEA-Cl reduces the growth amplitude (magnitude) parameter *C* only slightly. Nonetheless, in our case part of the observed growth can be accommodated in the diffusion rate (*k*_2_). The values of coefficient *D* for TEA-Cl and BaCl_2_ are at a comparable level to their control (see Table 3D). For comparison, the effect of Cl^−^ channel blockers (A-9-C and DIDS) on the diffusion rate (*k*_2_) was about two-fold smaller than the effect of the K^+^ channel blockers (TEA-Cl and BaCl_2_). However, the values of both anion channel blockers are slightly larger than the control in the APW.

It was shown in Fig. 1a, b (Burdach et al. 2014) that the replacement of KCl by KNO_3_ diminished endogenous growth. From our results, which are depicted in Tables 4C and 5C, we can see a similar behaviour for both substances, regardless of the concentration used. Furthermore, the authors state that the growth-stimulatory effect of IAA did not depend on KNO_3_, which is consistent with our results (Tables 4, 5). The fact that IAA stimulated growth two-fold is clearly shown not only in the parameter *C* but also in the diffusion rate (*k*_2_). In our Tables 4D and 5D the application of IAA, regardless of the concentration of KNO_3_, increased the value of the coefficient *D* almost two-fold, and that result is in agreement with the conclusions in Burdach et al. (2014). However, there is a discrepancy in the conclusions when KNO_3_ is replaced by KCl. The application of IAA for 1 mM KCl increases the diffusion rate *k*_2_ considerably. The value of *k*_2_ remains at the same level after treatment with 10 mM KCl, regardless of whether auxin has been applied or not.

Considering Fig. 4 in Burdach et al. (2014), the application of 5 mM of KCl plus 5 mM KNO_3_ is, in author’s opinion, evidence that the total IAA-induced growth of maize coleoptile segments depends specifically on chloride ions. The authors state that IAA induces practically the same growth response, regardless of the concentration of KCl (1 or 10 mM) that is used. What is interesting is that the amplitude parameter *C* for KCl treatment was also similar to the control in the APW (see Table 6C), although the replacement of KCl with KNO_3_ dramatically decreases this amplitude independent of whether KNO_3_ was used with or without IAA (endogenous growth).

Further, the authors say (Fig. 5 in Burdach et al. 2014) that their data revealed no differences between the growth of coleoptile segments that were incubated in a medium containing 10 mM KCl or 5 mM KCl plus 5 mM KNO_3_, thus suggesting that NO_3_^−^ ions did not inhibit IAA-induced growth in the presence of KCl. It can be deduced from Table 7C that the influence of 5 mM KCl plus 5 mM KNO_3_ in auxin-induced growth is similar to the control in the APW, and that the influence of their control is almost at the same level. Let us note that the smallest value of parameter *C* for 10 mM KNO_3_ plus IAA (Table 7) corresponds to the highest value of coefficient *D* in Table 7D. It seems that part of the observed growth was accommodated in the diffusion rate (*k*_2_) and coefficient *C*.

The authors assert (Fig. 6 in Burdach et al. 2014) that their data indicate that A-9-C and DIDS decreased the IAA-induced growth of coleoptile segments by 32 and 43%, respectively. TEA-Cl added to the incubation medium reduced IAA-induced growth by 50%. It can be inferred from Table 8C that all three channel blockers reduced the growth magnitude parameter *C* although it seems surprising that coefficient *C* for TEA-Cl is similar to DIDS. Even more surprising is the fact that the parameter values *D* for the all of the channel blockers that were used are comparable and that they are significantly higher than controls (Table 8D).

The result presented in Fig. 7 by Burdach et al. (2014) indicates that the inhibition of IAA-induced growth was similar to that observed for TEA-Cl only in the presence of both blockers (TEA-Cl plus A-9-C), which in similar to our case when considering the amplitude coefficient *C* in Table 9C. Also, it can be deduced that the changes in growth magnitude parameter *C* for 10 mM KCl with IAA is similar to both controls. The diffusion rate *k*_2_ illustrates the impact of TEA-Cl, which is at the same level as the A-9-C (see Table 9D) for the auxin-induced growth of maize coleoptile segments. Moreover, the authors assert that the experiments in which TEA-Cl and A-9-C were used simultaneously suggest a coupling between the transport of K^+^ and Cl^–^ ions; this conclusion remains imprecise.

Based on Fig. 8 in Burdach et al. (2014) the authors declare that the addition of A-9-C to a medium containing 10 mM KNO_3_ diminished the IAA-induced growth of maize coleoptile segments only slightly compared to the medium containing 10 mM KCl, which is consistent with our results for parameter *C* (Table 10C) and for the impact of 10 mM KNO_3_ plus IAA. However, part of the observed growth can be accommodated in the diffusion rate (*k*_2_).

In conclusion, the analysed data reveals an overall regularity. In the majority of cases, the small amplitude of parameter *C* indicates a high value of diffusion coefficient *k*_2_. When comparing the charts for the parameters *C* and *D* the reversed trend can usually be observed.

Biological interpretation for a coefficient *B* has not been established yet.

### Turgor pressure

The consideration of turgor pressure (the scenario for Cl^−^ uptake in the presence of IAA) that is presented in Burdach et al. (2014) is only based on the Shabala et al. (2000) and Shabala and Lew (2002) articles, and are not supported by any original data in Burdach’s paper. Thanks to the new methods (fitexex) that are introduced in our work, at least in some cases the preliminary data of the turgor pressure can be assessed with the help of *A* coefficient using the same raw growth data (Tables 2A, 3, 4, 5, 6, 7, 8, 9A and 10). Last but not least, this approach allows the claims that qualitatively ‘predicted’ the turgor pressure behaviour in elongating coleoptile segments to be quantitatively substantiated.

### Cross-correlation: elongation growth–pH

**Fig. 4:**
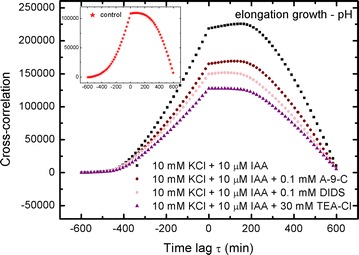
Cross-correlations of pH and elongation growth as a function of time lag τ (min). Analysed data for the insets in Fig. 6 (Burdach et al. 2014)

**Fig. 5:**
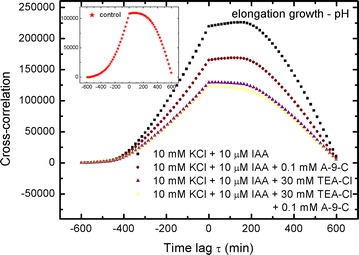
Cross-correlations of pH and elongation growth as a function of time lag τ (min). Analysed data for the insets in Fig. 7 (Burdach et al. 2014)

**Fig. 6:**
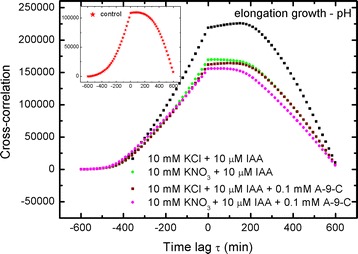
Cross-correlations of pH and elongation growth as a function of time lag τ (min). Analysed data for the insets in Fig. 8 (Burdach et al. 2014)

The results for cross-correlations between elongation growth and pH are shown in Figs. 2, 3, 4, 5 and 6. A similar situation can be observed in all of the charts—the remarkable regular shape of the cross-correlation curve for all experiments (originating from very irregular input data presented in Burdach et al. 2014) is almost the same irrespective of the treatments that was used, although the values differ. Furthermore, the time lag τ (delay) is either present or absent and is different for every sample. These results would certainly differ if abraded coleoptiles (Peters and Felle 1991) were used instead of coleoptile segments.

**Fig. 7:**
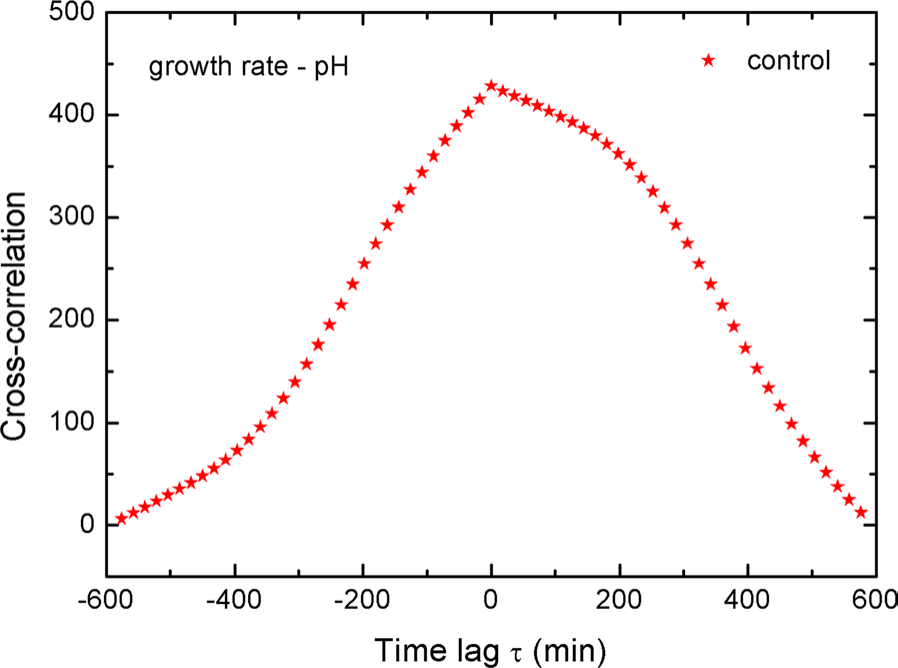
Calculated cross-correlation of growth rate and pH for control data (Rudnicka et al. 2014)

Our conclusions from the cross-correlation derivative are similar to the results that were obtained by Burdach et al. (2014) using electrophysiological methods. It seems that when using a cross-correlation for elongation growth, pH is the best way to present the time delay between these two processes. For comparison, we calculated the cross-correlation of the growth *rate* and pH for our control and present it in Fig. 7 (the original data was read from Fig. 1 in Rudnicka et al. 2014).

### pH measurement versus cross-correlation based H^+^-activity

**Fig. 8:**
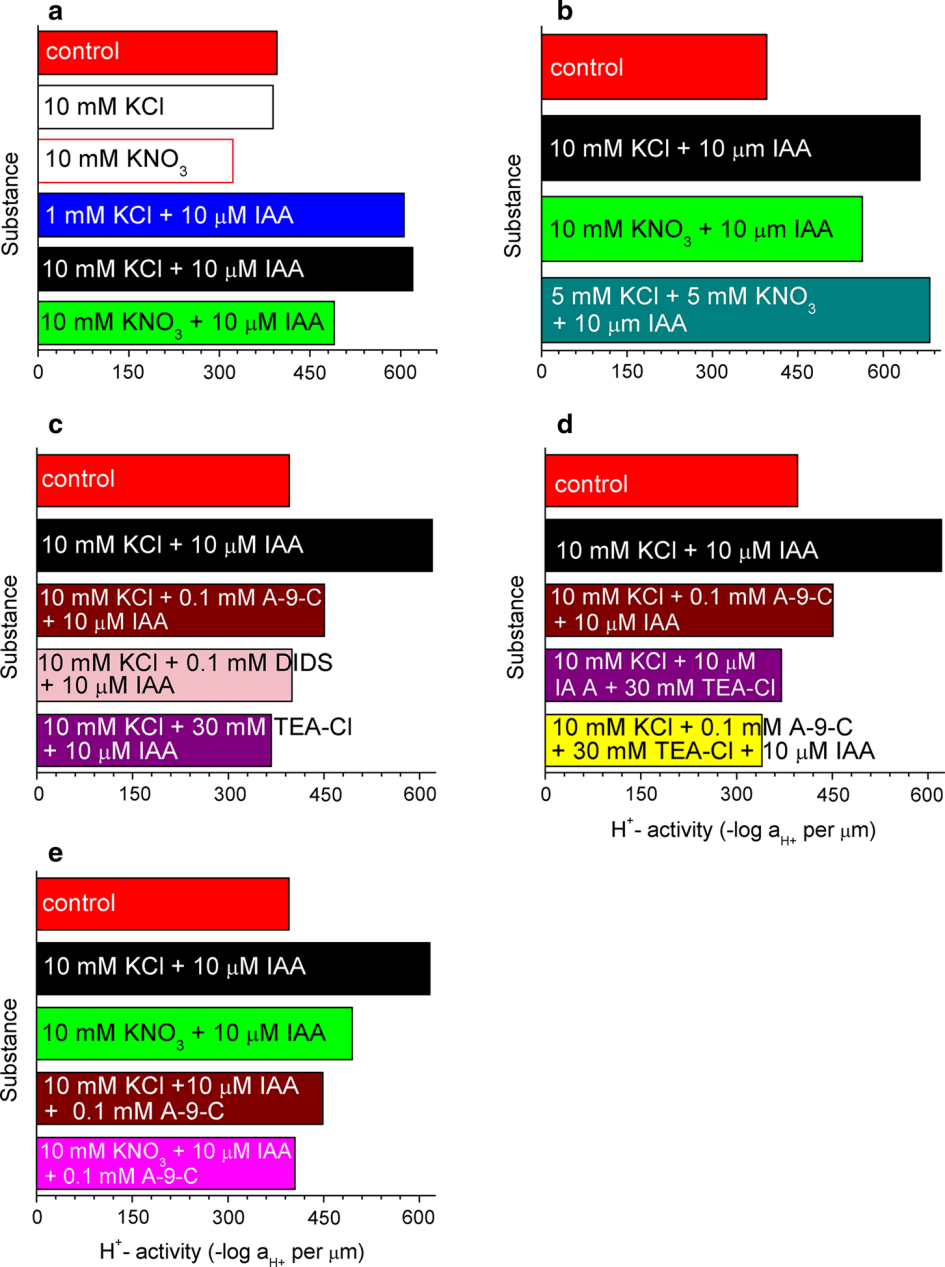
**a** IAA-induced changes (except for 10 mM KCl and 10 mM KNO_3_) in the acid secretion of the coleoptile segments of the model plant *Zea mays* L. calculated by the time derivative discontinuity in the cross-correlation. For further description, see the caption to Additional file 1: SI Fig. 10. **b** IAA-induced changes in the acid secretion of the coleoptile segments of the model plant *Zea mays* L. calculated by the time derivative discontinuity in the cross-correlation. For further description, see the caption to Additional file 1: SI Fig. 11. **c** The caption as in **a** and further description in Additional file 1: SI Fig. 12. **d** The caption as in **a** and further description in Additional file 1: SI Fig. 13. **e** The caption as in **a** and further description in Additional file 1: SI Fig. 14

In Burdach et al. (2014), it is stated that medium pH changes, which were measured simultaneously with growth, indicated that KNO_3_ inhibited both IAA-induced proton extrusion and proton extrusion in an auxin-free medium (Fig. 4, left inset, ibid.). Our results, which are presented in Fig. 8a, confirm the proton efflux by KNO_3_ for auxin-induced growth as well as for the pure control compared to their control. However, the application of an APW-based control allows new light to be shed on the acquired data. H^+^-activity for 10 mM KCl is similar to the APW-control, while the value for KNO_3_ is slightly lower. In turn, our observation that KCl in the presence of IAA, irrespective of the concentration (1 or 10 mM) used, maintains H^+^-activity at a similar level, are in contradiction to Burdach et al. (2014). In that work, which is based on Fig. 4 (left inset), it is claimed that proton extrusion was stimulated by auxin much more effectively at 10 mM KCl than at 1 mM KCl, thus supporting the hypothesis that auxin enhances the H^+^/K^+^ antiport at the plasma membrane. Our data are also in contradistinction to the statement that their data in Fig. 4 (right inset) indicate that the enhanced proton extrusion that was observed in the presence of IAA and 10 mM KCl did not necessarily result in the elongation growth of coleoptile segments being significantly greater than those in the medium with IAA and 1 mM KCl.

For whatever a comment deserve the results for pH presented in Fig. 5 in Burdach et al. (2014), entirely omitted by the authors. In Fig. 8a it can be clearly seen that the replacement of KCl by KNO_3_ in the IAA-induced growth of coleoptile segments caused a decrease in H^+^-activity, though the influence of auxin is still visible compared to the endogenous growth in the APW. In addition, from Fig. 8b it can deduced that, similar to the analysed article (Burdach et al. 2014), the KCl concentration, which decreased from 10 mM to 5 mM with the simultaneous addition of 5 mM KNO_3_, had no impact on the auxin-induced growth and H^+^-activity, even though changes were observed in pH.

Medium pH changes, which were measured simultaneously with growth (Fig. 6, left inset, ibid.), showed that while DIDS and TEA-Cl completely eliminated IAA-induced proton extrusion, A-9-C only diminished it by 50% (expressed as H^+^ concentration in the medium at 10 h). All three channel blockers (our Fig. 8c) caused an inhibition of H^+^-activity compared to control with 10 mM KCl plus 10 µM IAA. However, the application of 30 mM TEA-Cl lowered the H^+^-activity to below the control level in the APW.

In Burdach et al. (2014), it can be observed that medium pH, which was measured simultaneously with the growth of maize coleoptile segments, indicated that while DIDS at 0.1 mM completely eliminated IAA-induced proton extrusion, A-9-C at the same concentration diminished it by only 50%. The authors concluded that the plasma membrane H^+^-ATPase might be involved in Cl^−^ uptake. These conclusions are in accord with our results (Fig. 8c) for H^+^-activity at 0.1 mM DIDS, which in the concentration that was applied cancels the stimulating effect of auxin completely and it finally reaches the same value as the control in the APW. It is intriguing that Burdach et al. (2014) were able to determine this conclusion without comparing their data with the APW endogenous growth.

Further, the changes in the pH of the medium (Burdach et al. 2014), which were measured simultaneously with growth, indicated that the addition of both blockers together (TEA-Cl and A-9-C) caused a strong inhibition of IAA-induced proton extrusion, which is characteristic of the action of TEA-Cl (Fig. 7, left inset). Experiments in which TEA-Cl and A-9-C were added together suggest a coupling between the transport of K^+^ and Cl^−^ ions. The data of proton efflux that is presented in Fig. 8 in Burdach et al. (2014) are compatible with ours, which are presented in Fig. 8e. The inhibitory effect of the simultaneous application of TEA-Cl and A-9-C is greater than when these channel blockers are applied individually. Moreover, the effect of the simultaneous application even exceeds the stimulating influence of auxin on growth (compare with the APW control).

Based on Fig. 8 Burdach et al. (2014) state that the inhibitory effect of KNO_3_ on IAA-induced proton extrusion was somewhat lower in the presence of A-9-C. By comparing their data (left inset in Fig. 8) and our data in Fig. 8e, we may say that the modest pH decrease that was caused by the addition of A-9-C to the medium containing KNO_3_ with IAA and IAA plus A-9-C does not agree with the decrease in proton activity that we observed.

Summing up, our observations concerning H^+^-activity, which are based on our Fig. 8 and pH measurement data in Burdach et al. (2014), converge providing that the decrease in pH is accompanied by elongation growth. A jump in the derivative (Additional file 1: SI Figs. 10–14; Fig. 8a–e) might be connected with the hydrogen bond breaking and the creation of H^+^ ions, and as a result, the acidification of the medium, which was observed with a decrease in pH.

### Spectral analysis

We also showed the structure of the growth factors by means of the induced growth rate spectrum, which displayed the reciprocal power (intensity) dependence of diffusion coefficient for different substances: auxin—IAA and anion: A-9-C or DIDS or cation: TEA-Cl or BaCl_2_ channel blockers.

**Fig. 9:**
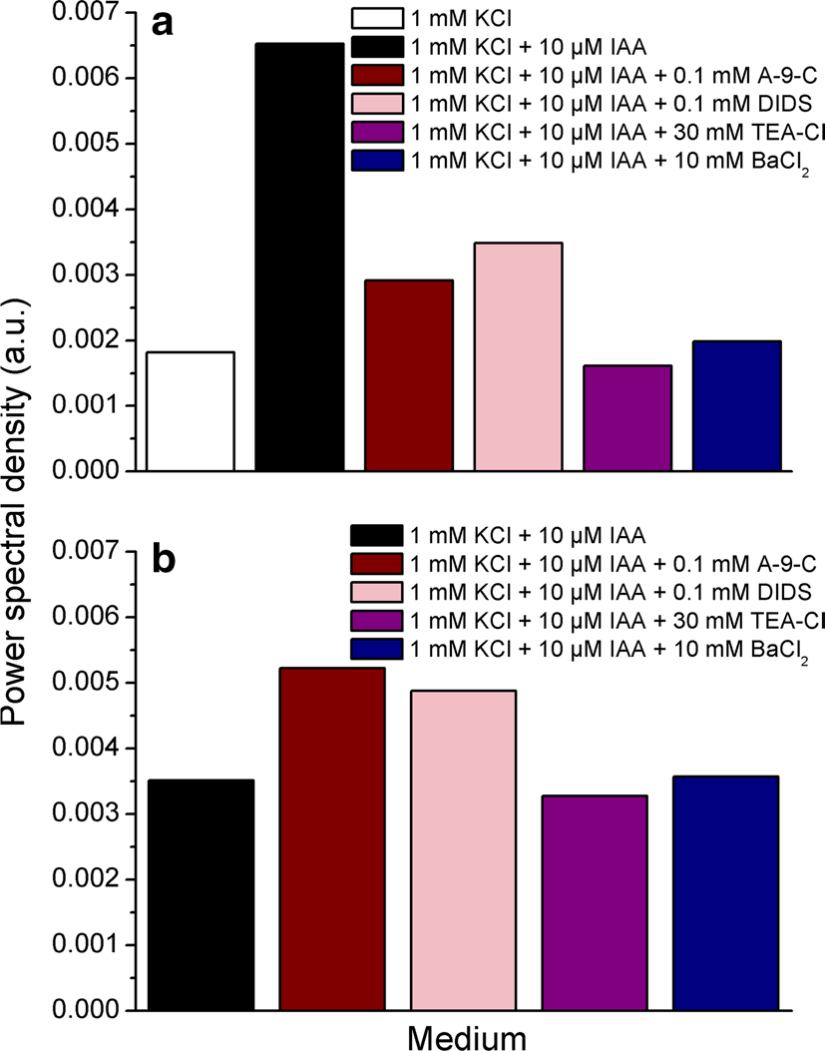
**a** Spectral power (intensity) response to different media: 1 mM KCl (*open square*), 1 mM KCl + 10 μM IAA (*closed square*), 1 mM KCl + 10 μM IAA + 0.1 mM A-9-C (*wine circle*), 1 mM KCl + 10 μM IAA + 0.1 mM DIDS (LT *magenta circle*), 1 mM KCl + 10 μM IAA + 30 mM TEA-Cl (*violet triangle*), 1 mM KCl + 10 μM IAA + 10 mM BaCl_2_ (*royal triangle*). The channel blockers were implemented after 1 h, then incubated in the presence of 10 μM IAA after 2 h. Data based on Additional file 1: SI Fig. 9A, corresponding to Eq. (9). **b** Spectral power (intensity) response to different media: 1 mM KCl + 10 μM IAA (*closed square*), 1 mM KCl + 10 μM IAA + 0.1 mM A-9-C (*wine circle*), 1 mM KCl + 10 μM IAA + 0.1 mM DIDS (LT *magenta circle*), 1 mM KCl + 10 μM IAA + 30 mM TEA-Cl (*violet triangle*), 1 mM KCl + 10 μM IAA + 10 mM BaCl_2_ (*royal triangle*). It was incubated in the presence of 10 μM IAA after 2 h, then channel blockers were implemented after 3 h. Data based on Additional file 1: SI Fig. 9B, corresponding to Eq. (9). The intensity corresponding to the power spectral density at 0 Hz

Additional file 1: SI Fig. 9A and B represent the Fourier analysis data from Figs. 2 and 3 in Burdach et al. (2014). The zero frequency mode (where elongation without oscillations takes place) for different treatments that we obtained are presented in Fig. 9a, b. Spectral density shows that IAA-induced growth processes in different media is strictly connected with the timing of the auxin application. The authors (ibid.) presented two alternative experiments: (1) Fig. 2—the channel blockers were added to the control medium after a 1 h preincubation and IAA was added to the incubation medium at 2 h and (2) auxin was added to the incubation medium at 2 h and the channel blockers 3 h later. Of these two variants, the first one seems to be better since the stimulating action of auxin on the growth and the inhibitive role of channel blockers are clearly visible.

### The derivative of elongation growth

**Fig. 10:**
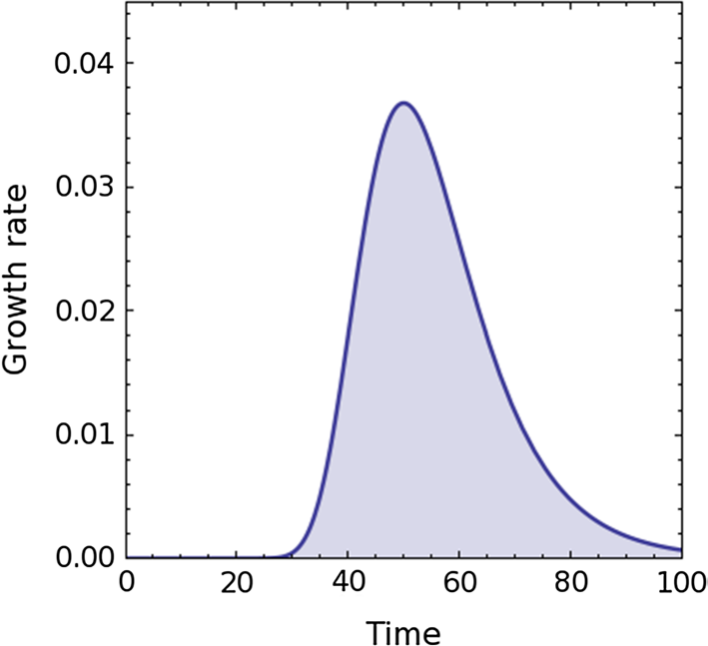
The growth rate f′(t) = C × D × exp[− exp(−D(t − t_e_) − D(t − t_e_))] calculated from the cumulative elongation of growth f(t) = C × exp[− exp(−D(t − t_e_))], Pietruszka (2012), as a function of time *t*. The inflection point *t*_e_ and *C*, *D* coefficients denote model free parameters: *C* for the dual (pH/T–dependent) growth amplitude (Pietruszka 2016) and *D* for the diffusion rate (not to be confused with the diffusion constant)

Additional file 1: SI Figs. 15–19 and Table 11 illustrate the derivative of the elongation growth where the location of the main peak, half-width and height is indicated. The area in these figures gives information about the total growth that can be used to draw further conclusions about the influence of the applied treatments. What is interesting is that the localization of the mean peak (except for the control in the APW, pure KCl or KNO_3_ treatment) seems to be almost in the same region at about 180 min (between 150 and 195 min). In our opinion, this fact may be connected with the response to the addition of IAA at 120 min of the experiment and the rapid increase of elongation in the first phase of this process. In addition, the shape of all of the charts (and growth rate plots in Figs. 4–8 in Burdach et al. 2014) that show the impact of IAA plus KCl or KNO_3_, with or without channels blockers, is the same as for the auxin-induced growth that was theoretically predicted by Pietruszka (2012) as is illustrated in Fig. 10 and Additional file 1: SI Figs. 15–19.

**Table 11:** Calculation of the mean total growth, total growth rate and the location of the main peak for different treatments of the data presented in Additional file 1: SI Figs. 15–19

Treatment	n	Total growth	Peak at	Total growth rate
10 mM KCl + 10 µM IAA	5	1787.72 ± 97.83	177 ± 12.6	8.33 ± 0.42
10 mM KNO_3_ + 10 µM IAA	3	1174.40 ± 90.47	180 ± 0	6.17 ± 0.44
10 mM KCl + 10 µM IAA + 0.1 mM A-9-C	3	1241.68 ± 5.23	180 ± 0	6.23 ± 0.07
10 mM KCl + 10 µM IAA + 30 mM TEA-Cl	2	910.23 ± 2.35	179 ± 10.6	5.09 ± 0.08

Errors calculated by SD

### The strength of the E_H+_ electrical field

The calculations preformed in Table 12, which are based on Additional file 1: SI Tables 1 and 2, deliver an important empirical relation

16 $$\hbox{E}_{\rm m} = \, \hbox{E}_{\rm H + } \cdot \left( {\hbox{log}_{10} 1/a_{\rm H + } \cdot \upmu {\text{m}}} \right)$$

where E_H+_ = 0.157 ± 0.009 [V/mm] for maize and E_m_ (mV) is the membrane potential in the perenchymal coleoptile cells of *Zea mays* L. Note that in Eq. (16) we have introduced a new constant (E_H+_). When this relation is known, the membrane potential E_m_ can be determined for intact plant growth or for different intervening substances (as it is re-examined in this article) *exclusively* from the growth (rate) and pH measurements. Apparently, the set of measurements that are usually performed together with the growth data can be reproduced *without* electrophysiological experiments, thus avoiding wasting resources.

**Table 12:** Calculation of the E_H+_–constant for the empirical formula E_H+_ = E_m_/(log_10_ 1/a_H+_ ∙ μm) = 0.157 ± 0.009, where E_m_ (mV) is the membrane potential in the perenchymal coleoptile cells of *Zea mays* L. (Additional file 1: SI Table 1) and H^+^-activity is taken from Additional file 1: SI Table 2

Treatment	E_m_ (mV)						
	A (0 min)	B (3 min)	C (6 min)	D (10 min)	E (20 min)	F (30 min)	Mean
1 mM KCl + IAA	−0.199	−0.200	−0.192	−0.194	−0.208	−0.217	−0.202 ± 0.009
10 mM KCl	−0.182	−0.177	−0.175	−0.175	−0.176	−0.175	−0.177 ± 0.003
10 mM KCl + IAA	−0.109 ± 0.011	−0.110 ± 0.011	−0.108 ± 0.011	−0.111 ± 0.009	−0.116 ± 0.010	−0.118 ± 0.011	−0.112 ± 0.004
10 mM KCl + IAA + A-9-C	−0.144 ± 0.009	−0.135 ± 0.009	−0.138 ± 0.008	−0.141 ± 0.010	−0.140 ± 0.007	−0.138 ± 0.008	−0.139 ± 0.003
Mean	−0.158	−0.155	−0.153	−0.155	−0.160	−0.162	−0.157 ± 0.009

Errors calculated using the total derivative and standard deviation methods

### Comment on the qualitative estimations of the impact of chloride ions on auxin-induced growth

The analysis presented by Burdach et al. (2014) should be clarified and strengthened through the use of analytical methods. Taking only recent theoretical outcomes like that of Barbacci et al. (2013) or Zajdel et al. (2016), would certainly deepen their analysis. More importantly, the more efficient methods that we used in this study should be introduced in further plant physiological analysis.

## Conclusions

A set of quite recent or new methods and is proposed, which will help in comparison of the data between groups using different experimental setups, on the basis of obtained results. Our work is accompanied by free and easy-to-use processing software—published recently by Zajdel et al. (2016)—which will help to propagate it among a wider audience. We strongly believe (ibid.) that in order to improve the understanding of the subject, the community needs to boost the transferability and comparability of the published results.

Our study has set off an unsettled controversy among plant physiologists as it still remains a matter of debate as to what extent auxin-induced cell wall acidification contributes to elongation growth (Kutschera and Edelmann 2005). Here, rather than expressing vague interpretations, we want to contribute to the elucidation of controversies that are escalating about the “acid growth hypothesis” with strict numbers that have been obtained by existing or newly developed techniques, and leaving the interpretations to the biologists who are working in the field.

### Main conclusion

A quantitative report on the impact of chloride on the kinetic coefficients of auxin-induced growth was produced using several novel analytical methods. A new constant, E_H+_ = –E_m_/(pH μm) = 0.157 ± 0.009 [V/mm], was found where E_m_ is membrane potential.

## Additional file

**Additional file 1.** Supplementary information.
