# Supplementary material for: A quantitative report on the impact of chloride on the kinetic coefficients of auxin-induced growth: a numerical contribution to the “acid growth hypothesis”
Source: Springerplus. 2016 Nov 15;5(1):1978. doi: 10.1186/s40064-016-3626-y (PMC5108741; doi:10.1186/s40064-016-3626-y)
Supplement: Supplementary file 1 — Additional file 1. Supplementary information. [file 40064_2016_3626_MOESM1_ESM.doc]

**SUPPLEMENTARY INFORMATION**

A quantitative report on the impact of chloride on the kinetic coefficients of auxin-induced growth. A numerical contribution to the “acid growth hypothesis”

Mariusz Pietruszka and Aleksandra Haduch-Sendecka

Plant Physiology, Faculty of Biology and Environment Protection, University of Silesia, Katowice, Poland

**Tables legends**

**SI Table 1** Membrane potential values as presented in Table 1 by Burdach et al. (2014). Errors calculated by the standard deviation method.

**SI Table 2** Author’s results for H^+^-activity (–log a_H+_ per μm) calculated by cross-correlation derivative, see also SI Figs 10 – 14. Errors calculated by the standard deviation method.

**SI Table 3** Cross-reference table, to aid the reader.

**Figures legends**

**SI Figure 1A** Fit to the data for control (closed diamond) of Polak et al. 2012 (Fig. 2) describing growth for coleoptile segments of maize under constant dim green light at 25⁰C. **B** Fit to the data for control (open square) of Rudnicka et al. 2014 (Fig. 1) describing growth or coleoptile of maize grown under constant dim green light at 25⁰C.

**SI** **Figure 2A** Fit to the data reconstructed in Fig. 1A that describes the growth for the coleoptile of maize grown under constant dim green light at 25 °C and the influence of anion (A-9-C and DIDS) and cation (TEA-Cl and BaCl_2_) channel blockers that were implemented after 1 h, then incubated in the presence of 10 μM IAA after 2 h. The strains used were: 1 mM KCl (open square), 1 mM KCl + 10 μM IAA (closed square), 1 mM KCl + 10 μM IAA + 0.1 mM A-9-C (open circle), 1 mM KCl + 10 μM IAA + 0.1 mM DIDS (closed circle), 1 mM KCl + 10 μM IAA + 30 mM TEA-Cl (open triangle), 1 mM KCl + 10 μM IAA + 10 mM BaCl_2_ (closed triangle); x-axis label in seconds (system SI is required to receive proper values of coefficients by fitexex). **B** Fit to the data reconstructed in Fig. 2A growth for the coleoptile of maize grown under constant dim green light at 25 °C and the influence of anion (A-9-C and DIDS) and cation (TEA-Cl and BaCl_2_) channel blockers, that were incubated in presence of 10 μM IAA after 2 h, then channel blockers implemented after 3 h. The strains used were: 1 mM KCl + 10 μM IAA (closed square), 1 mM KCl + 10 μM IAA + 0.1 mM A-9-C (open circle), 1 mM KCl + 10 μM IAA + 0.1 mM DIDS (closed circle), 1 mM KCl + 10 μM IAA + 30 mM TEA-Cl (open triangle), 1 mM KCl + 10 μM IAA + 10 mM BaCl_2_ (closed triangle).

**SI Figure 3** Fit to the data of Burdach et al. 2014 (Fig. 1) describing growth for coleoptile of maize grown under constant dim green light at 25 °C and influence of KCl or KNO_3_. The strains used as follows: 1 mM KCl (open square), 1 mM KNO_3_ (open circle), 1 mM KCl + 10 μM IAA (closed square), 10 mM KNO_3_ + 10 μM IAA (closed circle) (**A**) and 10 mM KCl (open square), 10 mM KNO_3_ (open circle), 10 mM KCl + 10 μM IAA (closed square), 10 mM KNO_3_ + 10 μM IAA (closed circle) (**B**).

**SI Figure 4** Fit to the data presented in Fig. 4, ibid., describing growth for coleoptile of maize grown under constant dim green light at 25 °C and influence of KCl or KNO_3_. The strains used as follows: 10 mM KCl (open square), 10 mM KNO_3_ (open circle), 1 mM KCl + 10 μM IAA (closed triangle), 10 mM KCl + 10 μM IAA (closed square), 10 mM KNO_3_ + 10 μM IAA (closed circle).

**SI Figure 5** Fit to the data presented in Fig. 5, ibid., describing growth for coleoptile of maize grown under constant dim green light at 25 °C and influence of KCl or KNO_3_. The strains used as follows: 10 mM KCl + 10 μM IAA (closed square), 10 mM KNO_3_ + 10 μM IAA (closed circle), 5 mM KCl + 5 mM KNO_3_ + 10 μM IAA (closed triangle).

**SI Figure 6** Fit to the data presented in Fig. 6, ibid., describing growth for coleoptile of maize grown under constant dim green light at 25 °C and influence of anion or cation channel blockers. The strains used as follows: 10 mM KCl + 10 μM IAA (closed square), 10 mM KCl + 0.1 mM A-9-C + 10 μM IAA (open circle), 10 mM KCl + 30 mM TEA-Cl + 10 μM IAA (closed circle), 10 mM KCl + 30 mM TEA-Cl + 10 μM IAA (open triangle).

**SI Figure 7** Fit to the data presented in Fig. 7, ibid., describing growth for coleoptile of maize grown under constant dim green light at 25 °C and influence of A-9-C and TEA-Cl. The strains used as follows: 10 mM KCl + 10 μM IAA (closed square), 10 mM KCl + 0.1 mM A-9-C + 10 μM IAA (open circle), 10 mM KCl + 30 mM TEA-Cl + 10 μM IAA (open triangle), 10 mM KCl + 0.1 mM A-9-C + 30 mM TEA-Cl + 10 μM IAA (closed circle).

**SI Figure 8** Fit to the data presented in Fig. 8, ibid., describing growth for coleoptile of maize grown under constant dim green light at 25 °C and influence of anion channel blocker and KCl or KNO_3_ plus IAA. The strains used as follows: 10 mM KCl + 10 μM IAA (closed square), 10 mM KNO_3_ + 10 μM IAA (closed circle), 10 mM KCl + 0.1 mM A-9-C + 10 μM IAA (open square), 10 mM KNO_3_ + 0.1 mM A-9-C + 10 μM IAA (open circle).

**SI Figure 9A** Power spectral density calculated from the data presented in Fig. 2, ibid.: 1 mM KCl (open square), 1 mM KCl + 10 μM IAA (closed square), 1 mM KCl + 10 μM IAA + 0.1 mM A-9-C (wine circle), 1 mM KCl + 10 μM IAA + 0.1 mM DIDS (LT magenta circle), 1 mM KCl + 10 μM IAA + 30 mM TEA-Cl (violet triangle), 1 mM KCl + 10 μM IAA + 10 mM BaCl_2_ (royal triangle). **B** Power spectral density from the data presented in Fig. 3, ibid.: 1 mM KCl + 10 μM IAA (closed square), 1 mM KCl + 10 μM IAA + 0.1 mM A-9-C (wine circle), 1 mM KCl + 10 μM IAA + 0.1 mM DIDS (LT magenta circle), 1 mM KCl + 10 μM IAA + 30 mM TEA-Cl (violet triangle), 1 mM KCl + 10 μM IAA + 10 mM BaCl_2_ (royal triangle).

**SI Figure 10** IAA – induced changes in acid secretion of the coleoptile segments of the model plant *Zea mays* L. indicated by a red and brown (for control) vertical line (time derivative discontinuity). Both magnitudes receive their accurate values specified in the chart. Cross-correlation derivative as a function of time lag for the action of 10 mM KCl (open square), 10 mM KNO_3_ (open pink circle), 1 mM KCl + 10 μM IAA (closed blue triangle), 10 mM KCl + 10 μM IAA (closed black square), 10 mM KNO_3_ + 10 μM IAA (closed green circle), see also Fig. 2.

**SI Figure 11** See SI Fig. 9 caption for full description. Cross-correlation derivative as a function of time lag for the action of 10 mM KCl + 10 μM IAA (closed black square), 10 mM KNO_3_ + 10 μM IAA (closed green circle), 5 mM KCl + 5 mM KNO_3_ + 10 μM IAA (closed dark cyan triangle), see Fig. 3.

**SI Figure 12** See SI Fig. 9 caption for full description. Cross-correlation derivative as a function of time lag for the action of 10 mM KCl + 10 μM IAA (closed black square), 10 mM KCl + 0.1 mM A-9-C + 10 μM IAA (close wine circle), 10 mM KCl + 30 mM TEA-Cl + 10 μM IAA (closed LT magenta circle), 10 mM KCl + 30 mM TEA-Cl + 10 μM IAA (closed violet triangle), see Fig. 4.

**SI Figure 13** See SI Fig. 9 caption for full description. Cross-correlation derivative as a function of time lag for the action of 10 mM KCl + 10 μM IAA (closed black square), 10 mM KCl + 0.1 mM A-9-C + 10 μM IAA (closed wine circle), 10 mM KCl + 30 mM TEA-Cl + 10 μM IAA (closed violet triangle), 10 mM KCl + 0.1 mM A-9-C + 30 mM TEA-Cl + 10 μM IAA (closed yellow circle), see Fig. 5.

**SI Figure 14** See SI Fig. 9 caption for full description. Cross-correlation derivative as a function of time lag for the action of 10 mM KCl + 10 μM IAA (closed black square), 10 mM KNO_3_ + 10 μM IAA (closed green circle), 10 mM KCl + 0.1 mM A-9-C + 10 μM IAA (closed wine square), 10 mM KNO_3_ + 0.1 mM A-9-C + 10 μM IAA (closed magenta circle), see Fig. 6.

**SI Figure 15 – 19** Calculated derivative of elongation growth (= growth rate) for different intervening substances (see auxiliary Table 10 in the main text). The area presents the total elongation, while the peak value is corresponding to the maximum elongation rate.

SI Table 1

| Burdach et al. 2014 | A (0 min) | B (3 min) | C (6 min) | D (10 min) | E (20 min) | F (30 min) |
| --- | --- | --- | --- | --- | --- | --- |
| 1 mM KCl | -120.1 ± 4.3 | -119.8 ± 3.9 | -119.2 ± 4.6 | -120.5 ± 5.8 | -121.0 ± 5.4 | -122.4 ± 6.1 |
| 1 mM KCl + IAA | -120.6 ± 7.4 | -121.0 ± 6.6 | -116.6 ± 4.9 | -117.3 ± 5.2 | -126.1 ± 6.7 | -131.3 ± 7.5 |
| 1 mM KCl + IAA + A-9-C | -108.4 ± 4.7 | -107.9 ± 5.4 | -105.4 ± 6.2 | -108.1 ± 5.2 | -115.8 ± 6.1 | -118.5 ± 6.8 |
| 10 mM KCl | -70.6 ± 4.1 | -68.8 ± 3.9 | -67.9 ± 4.3 | -68.2 ± 4.5 | -68.4 ± 3.8 | -67.9 ± 4.2 |
| 10 mM KCl + IAA | -68.7± 4.5 | -69.2 ± 4.7 | -67.8 ± 3.8 | -70.0 ± 3.4 | -72.8 ± 4.1 | -74.1 ± 4.3 |
| 10 mM KCl + IAA + A-9-C | -64.8 ± 3.9 | -60.8 ± 4.2 | -62.4 ± 3.6 | -63.6 ± 4.5 | -63.1 ± 3.3 | -62.2 ±3.5 |

SI Table 2

| Author’s results | SI Fig. 10 | SI Fig. 11 | SI Fig. 12 | SI Fig. 13 | SI Fig. 14 | **mean** ± SD |
| --- | --- | --- | --- | --- | --- | --- |
| Burdach et al. 2014 | Fig. 4 | Fig. 5 | Fig. 6 | Fig. 7 | Fig. 8 |  |
| 1 mM KCl + IAA | 605.89 |  |  |  |  | **605.90** |
| 10 mM KCl | 388.70 |  |  |  |  | **388.70** |
| 10 mM KCl + IAA | 620.22 | 664.13 | 619.61 | 620.80 | 616.39 | **628.23** ± 20.14 |
| 10 mM KCl + IAA + A-9-C |  |  | 450.35 | 450.90 |  | **450.60** ± 0.38 |

**SI Table 3**

| *Burdach et al. 2014* | *Article* | | | | | | *Supporting Information* | | | |
| --- | --- | --- | --- | --- | --- | --- | --- | --- | --- | --- |
|  | Elongation growth | Coefficients *A* - *D* | Cross-correlation elongation growth – pH | Cross correlation growth rate - pH* | H^+^-activity | Power spectral density | Fits from fitexex | Power spectral density | Cross-correlation derivatives | Derivative of elongation growth |
| F1 |  | Table 4, 5 |  | Fig. 7* |  |  | *SI Fig. 3* |  |  |  |
| F2 | Fig. 1A,  *SI Fig. 2A* | Table 2 |  |  |  | Fig. 9A |  | *SI Fig. 9A* |  |  |
| F3 | Fig. 1B,  *SI Fig. 2B* | Table 3 |  |  |  | Fig. 9B |  | *SI Fig. 9B* |  |  |
| F4 |  | Table 6 | Fig. 2 |  | Fig. 8A |  | *SI Fig. 4* |  | *SI Fig 10* | *SI Fig 15* |
| F5 |  | Table 7 | Fig.3 |  | Fig. 8B |  | *SI Fig. 5* |  | *SI Fig. 11* | *SI Fig. 16* |
| F6 |  | Table 8 | Fig. 4 |  | Fig. 8C |  | *SI Fig. 6* |  | *SI Fig. 12* | *SI Fig. 17* |
| F7 |  | Table 9 | Fig. 5 |  | Fig. 8D |  | *SI Fig. 7* |  | *SI Fig. 13* | *SI Fig. 18* |
| F8 |  | Table 10 | Fig. 6 |  | Fig. 8E |  | *SI Fig. 8* |  | *SI Fig. 14* | *SI Fig. 19* |

Legend:

F - original figures from other authors

Fig - figures in our article

*data from Rudnicka et al. 2014: Fig. 1 for comparison

| control | | *Article* | *SI* |
| --- | --- | --- | --- |
| Polak et al. 2012 | F2 | Table 1 | *SI Fig. 1A* |
| Rudnicka et al. 2014 | F1 | Table 1 | *SI Fig. B 1* |

**
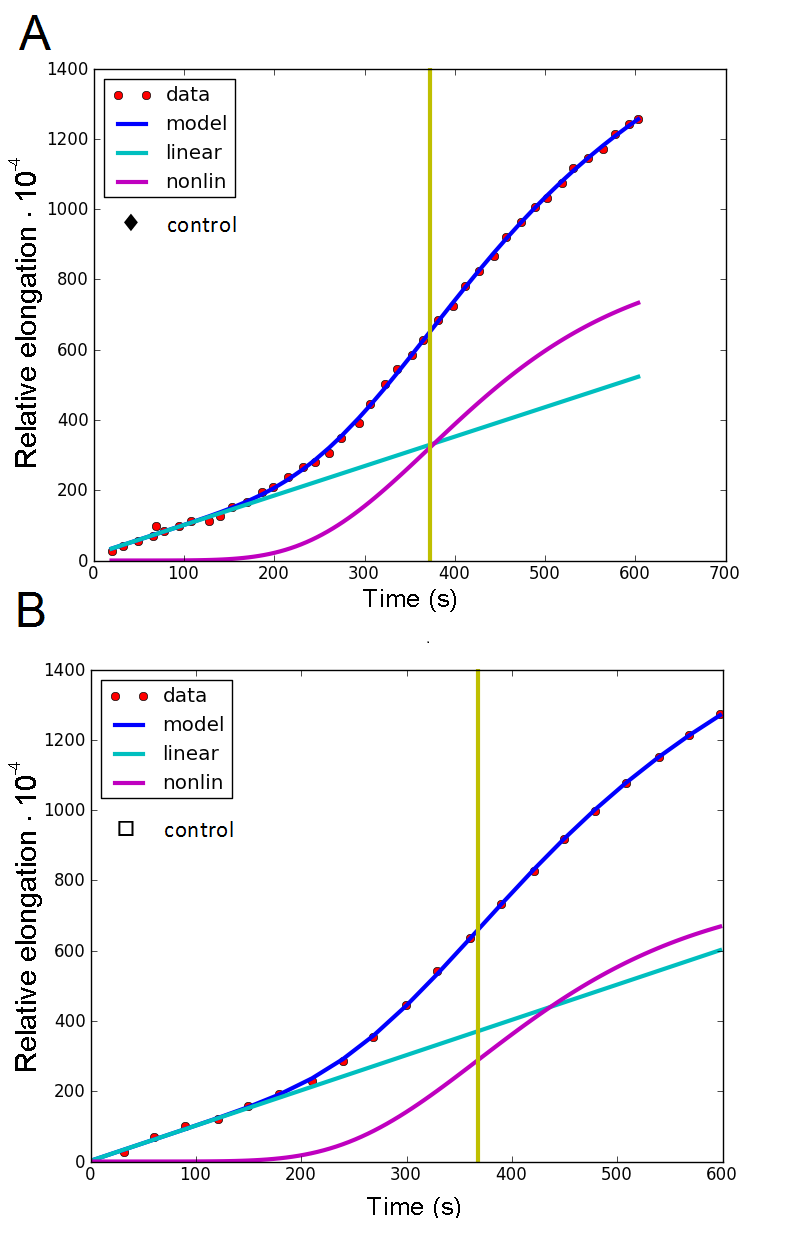
**

**SI Figure 1**

**
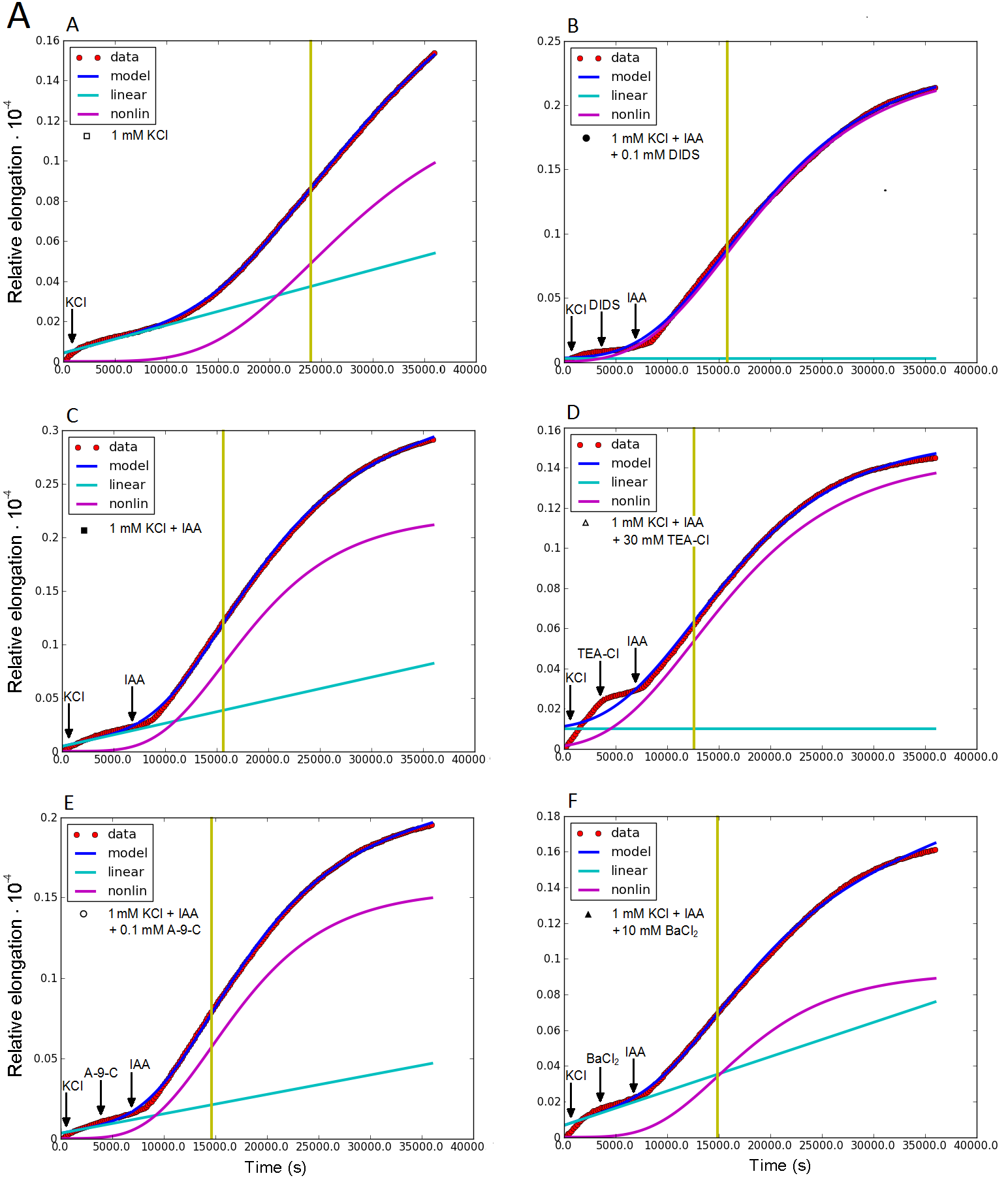
**

**SI Figure 2A**

**
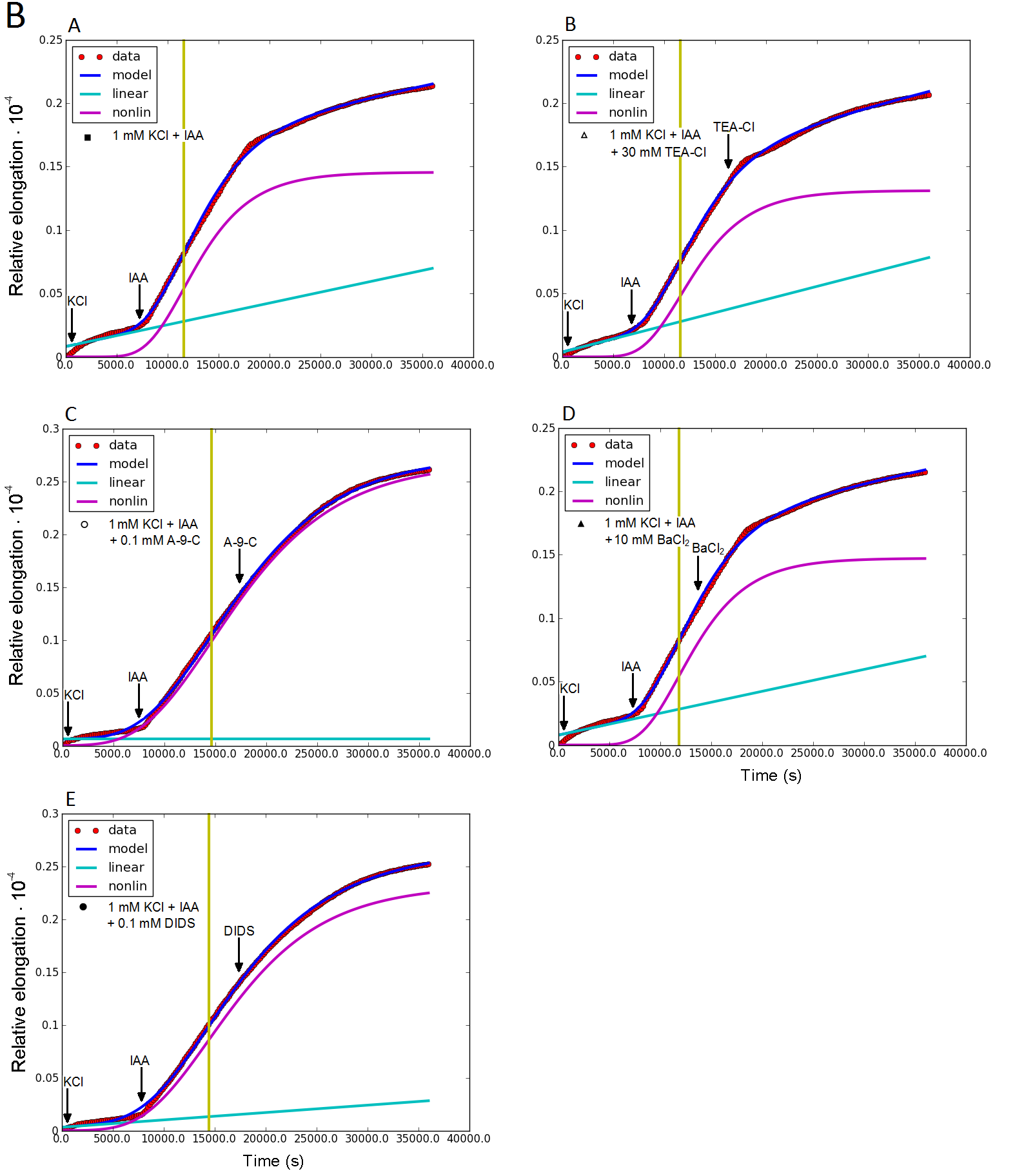
**

**SI Figure 2B**

**
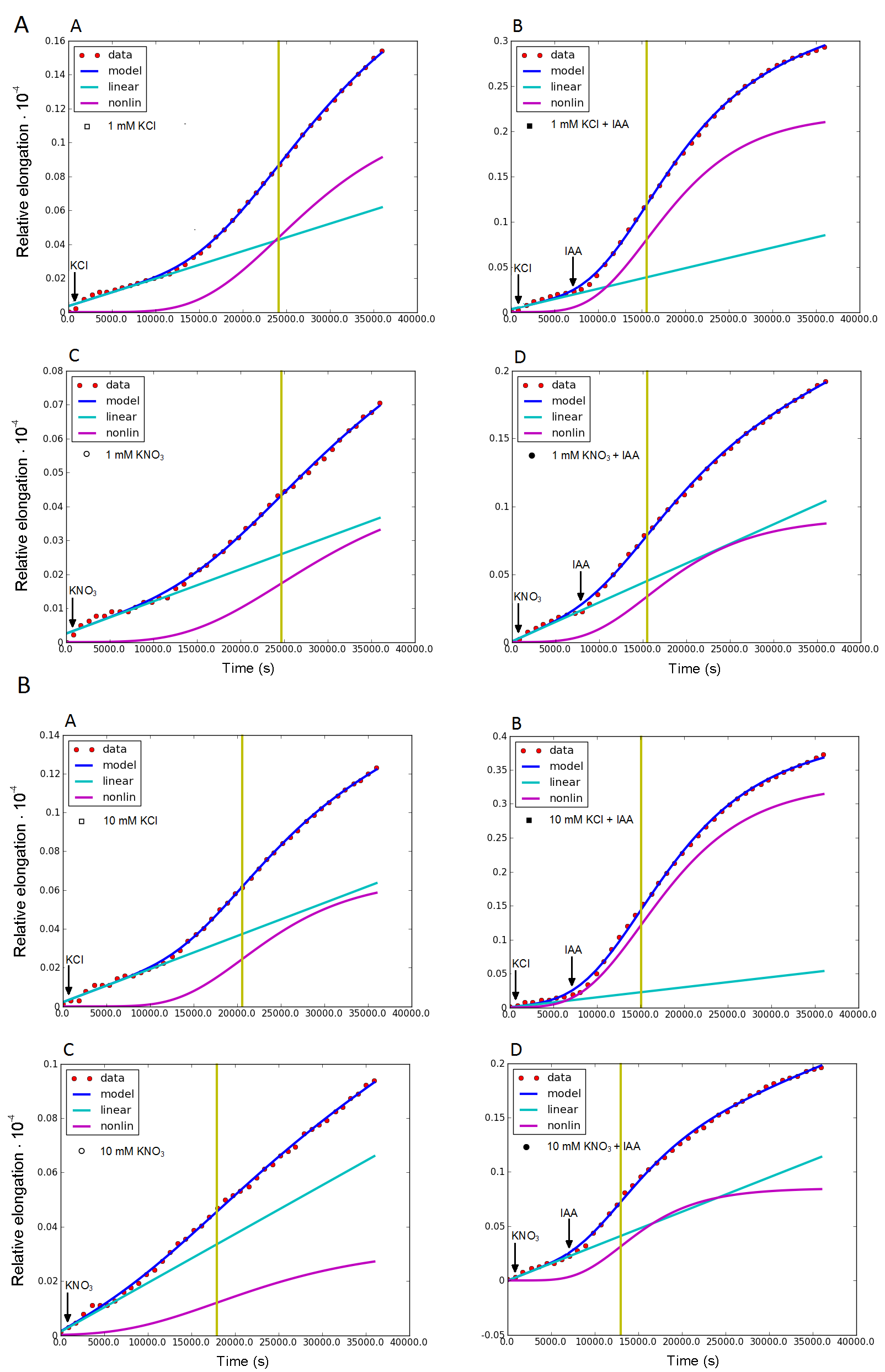
**

**SI Figure 3**

**
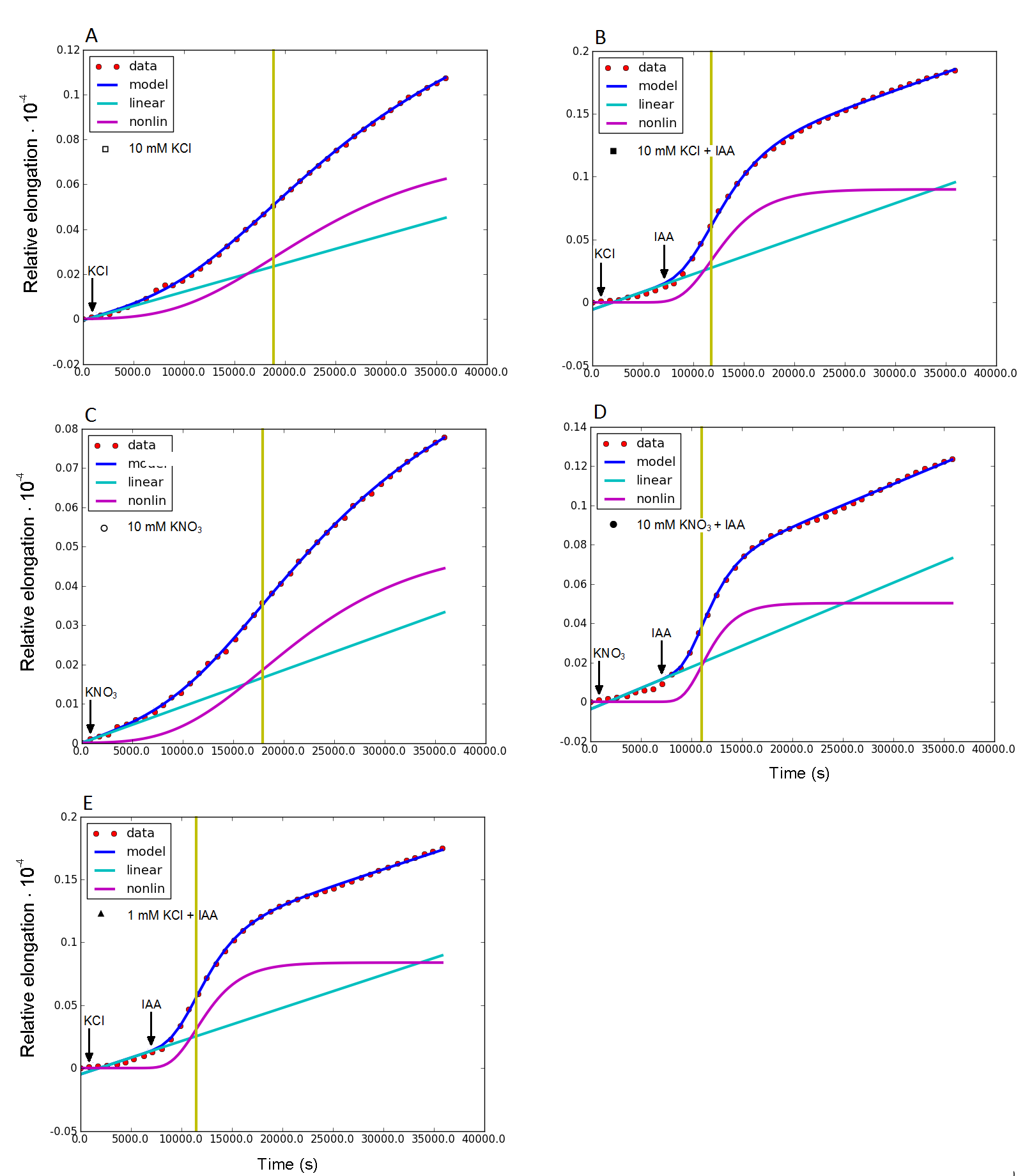
**

**SI Figure 4**

**
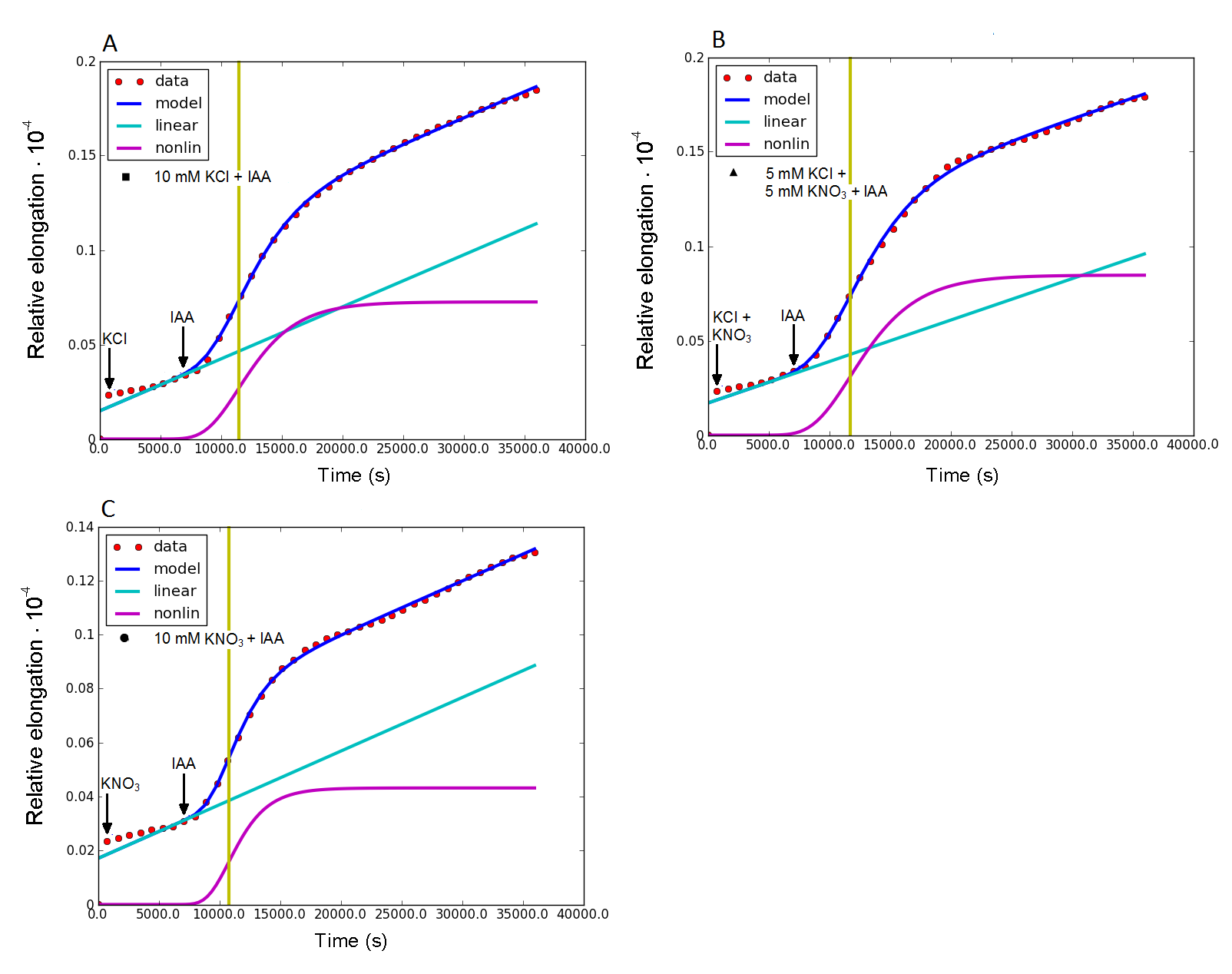
**

**SI Figure 5**

**
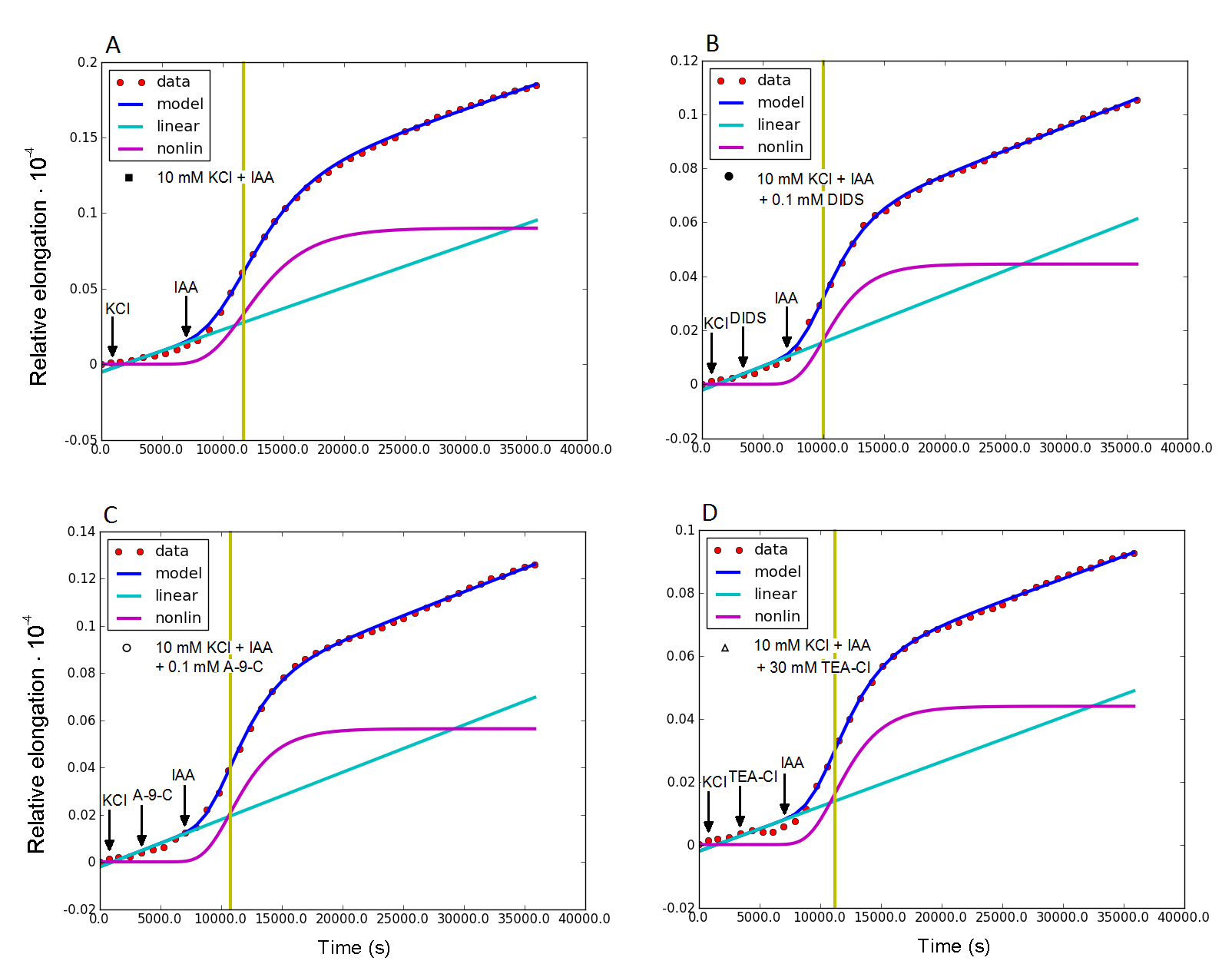
**

**SI Figure 6**

**
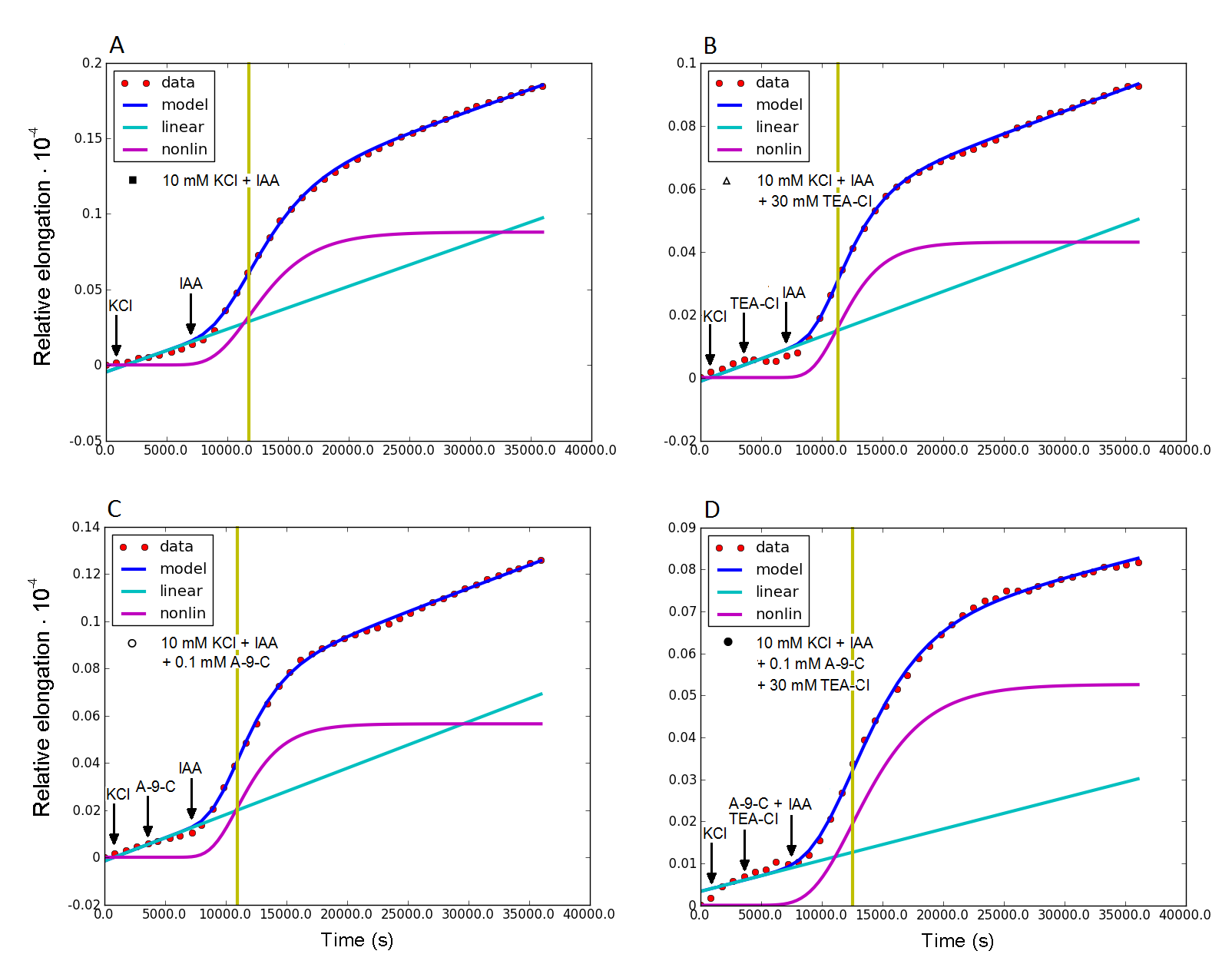
**

**SI Figure 7**

**
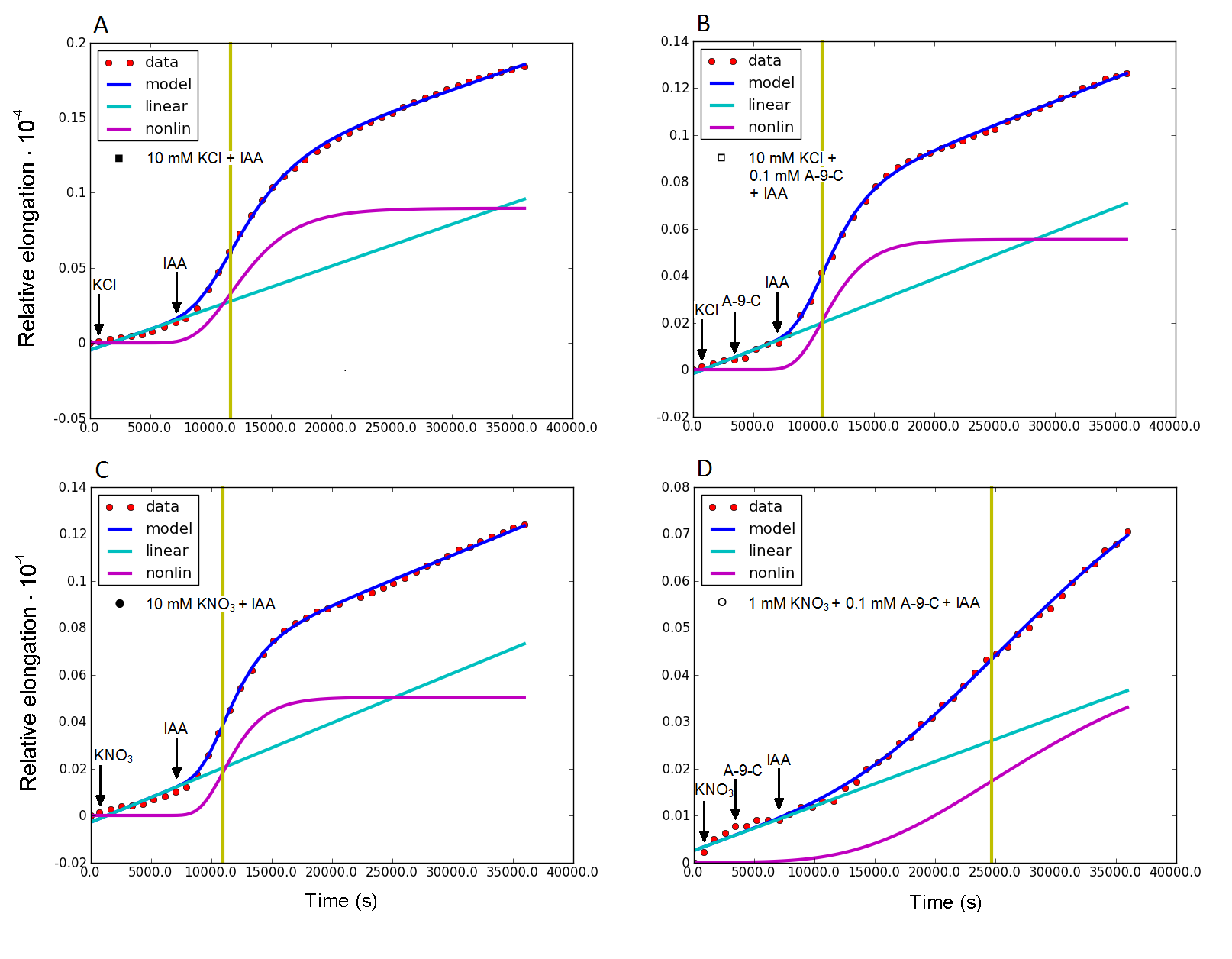
**

**SI Figure 8**

**
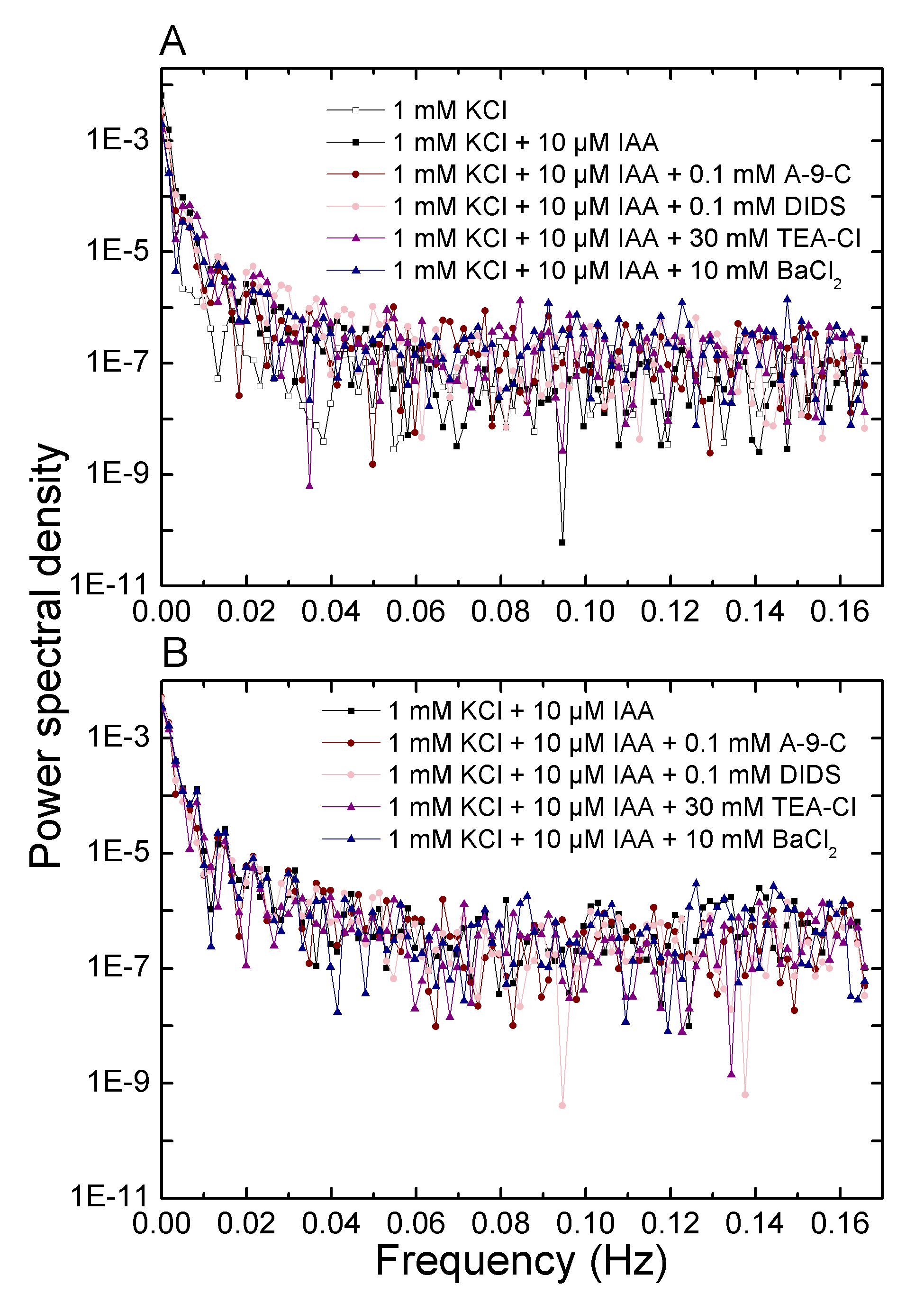
**

**SI Figure 9**

**
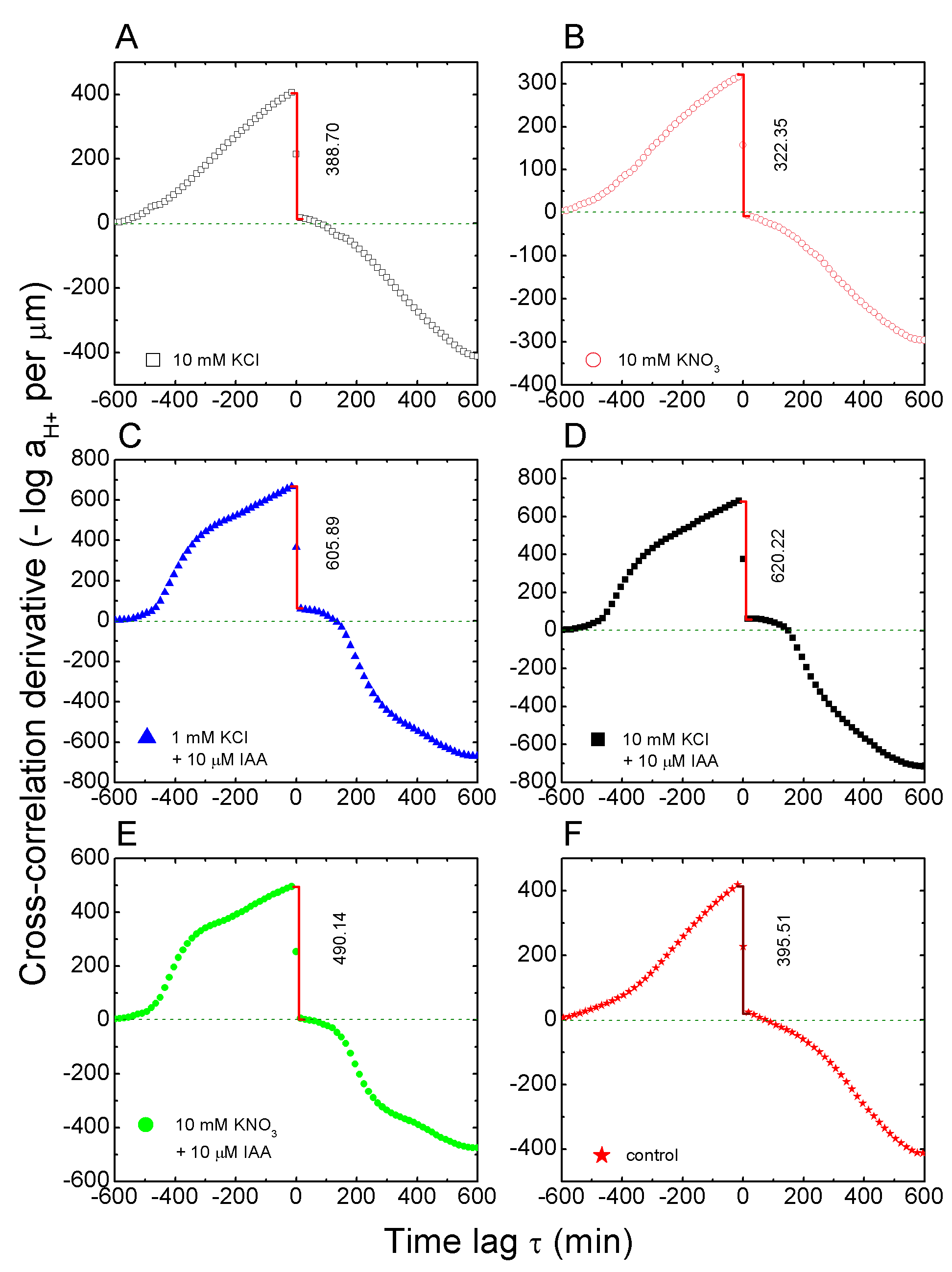
**

**SI Figure 10**

**
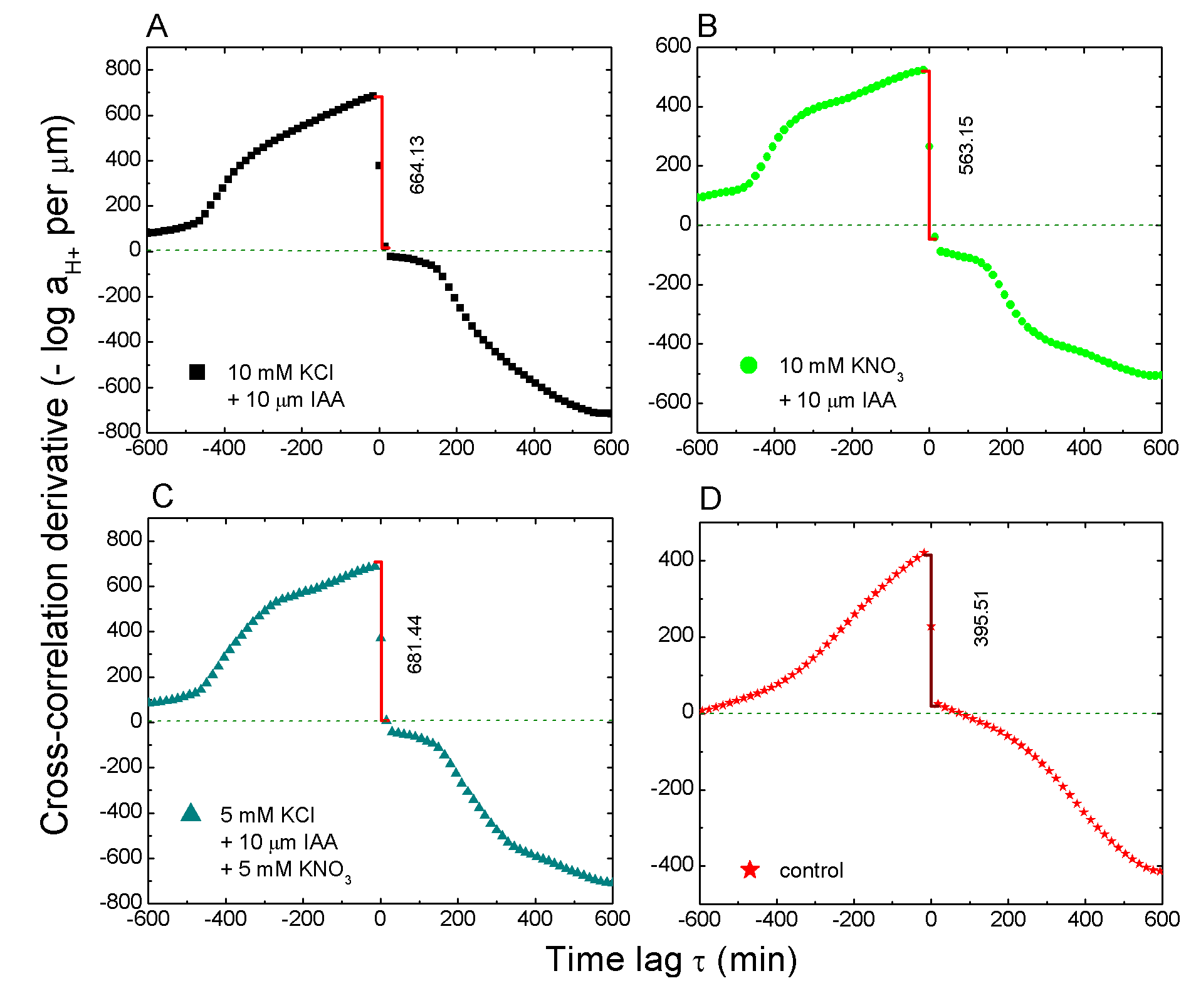
**

**SI Figure 11**

**
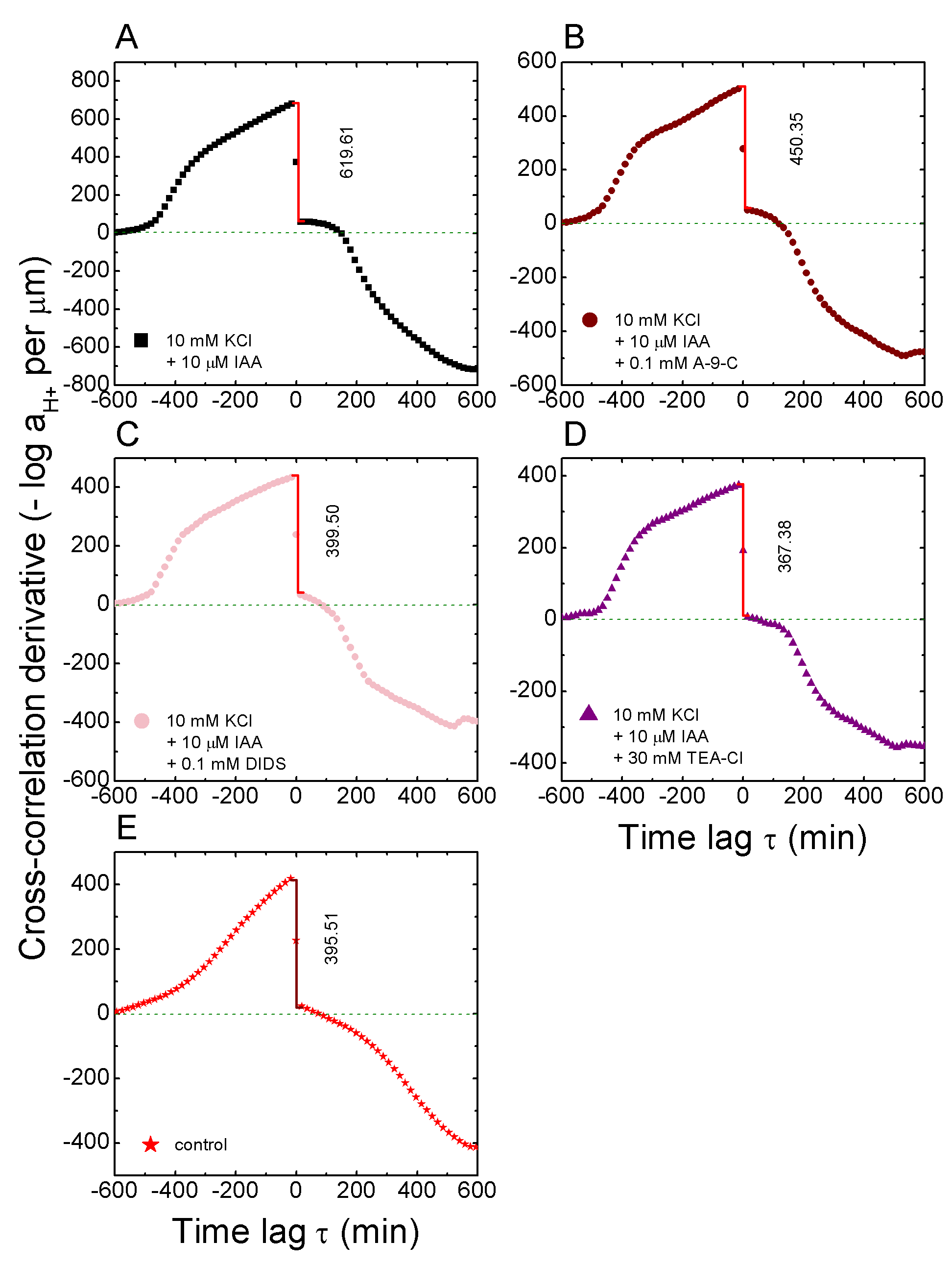
**

**SI Figure 12**

**
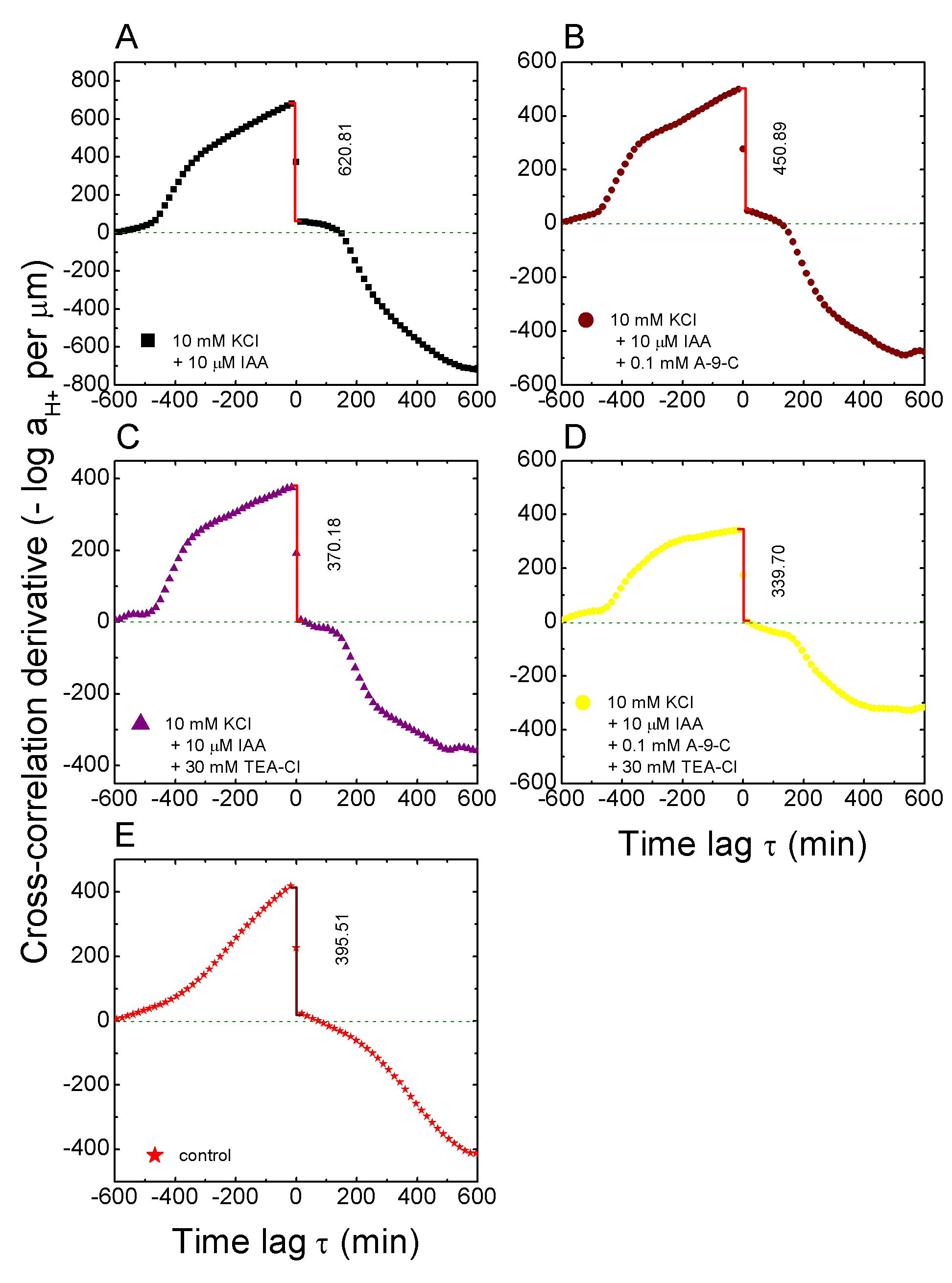
**

**SI Figure 13**

**
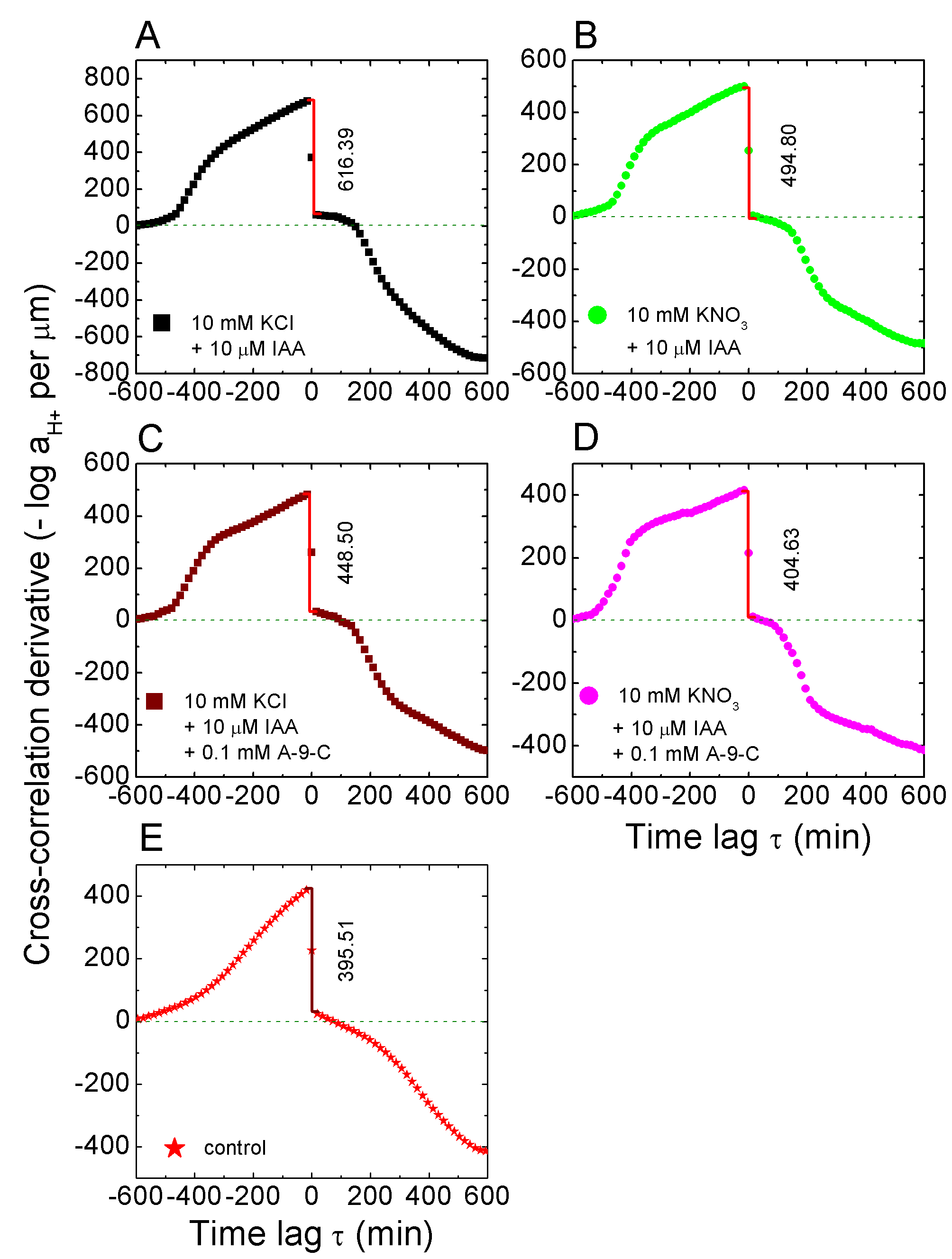
**

**SI Figure 14**

**
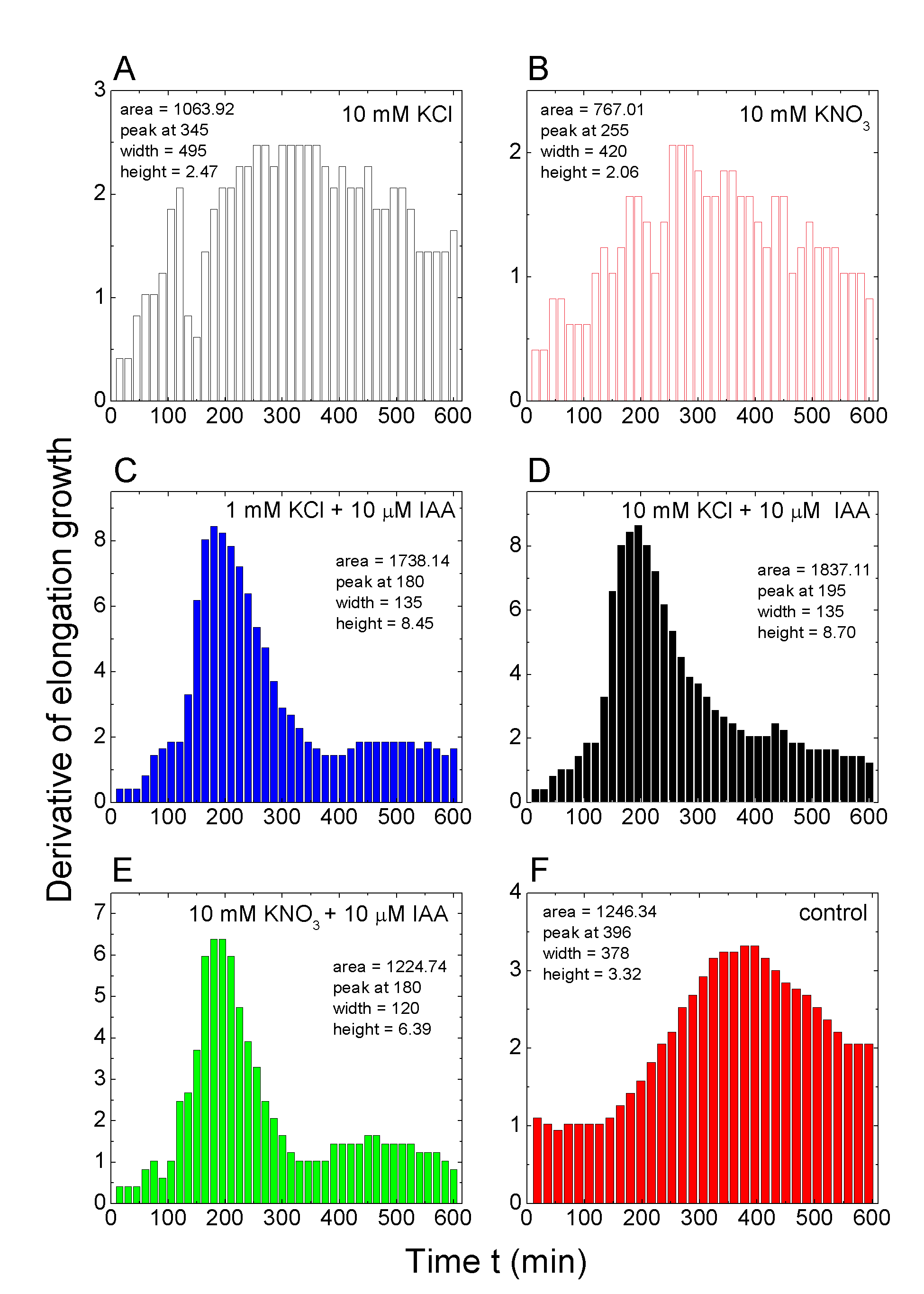
**

**SI Figure 15**

**
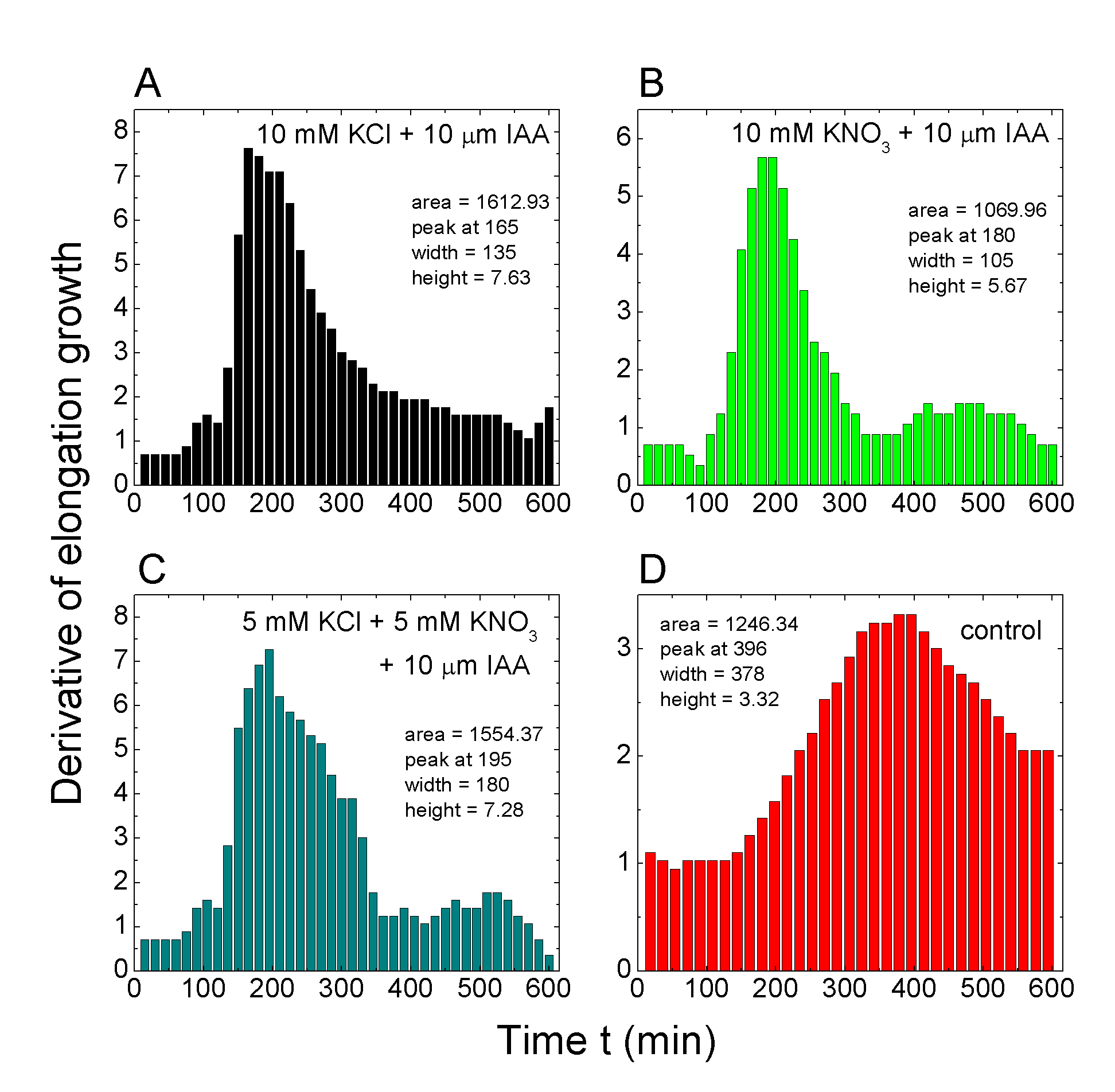
**

**SI Figure 16**

**
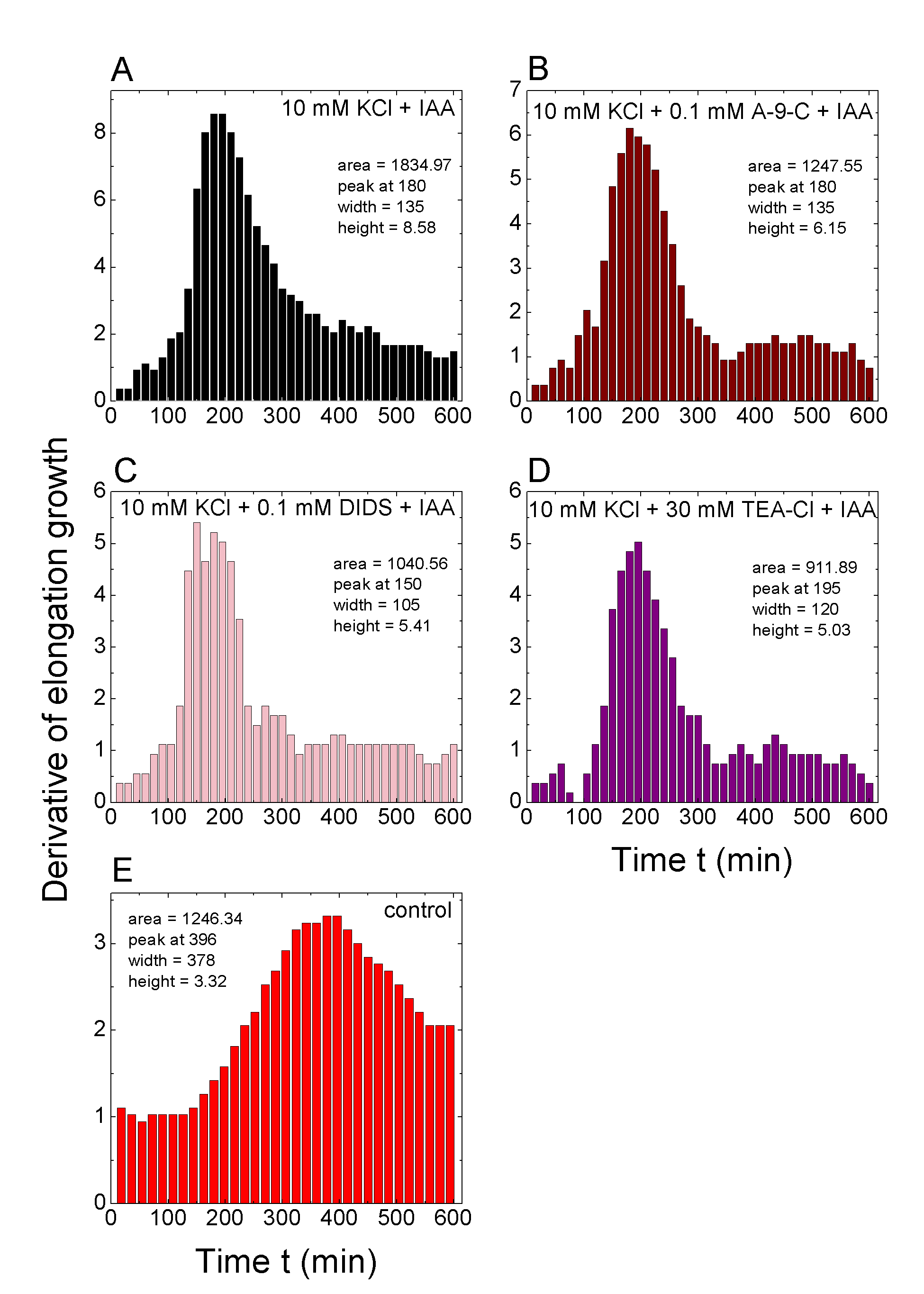
**

**SI Figure 17**

**
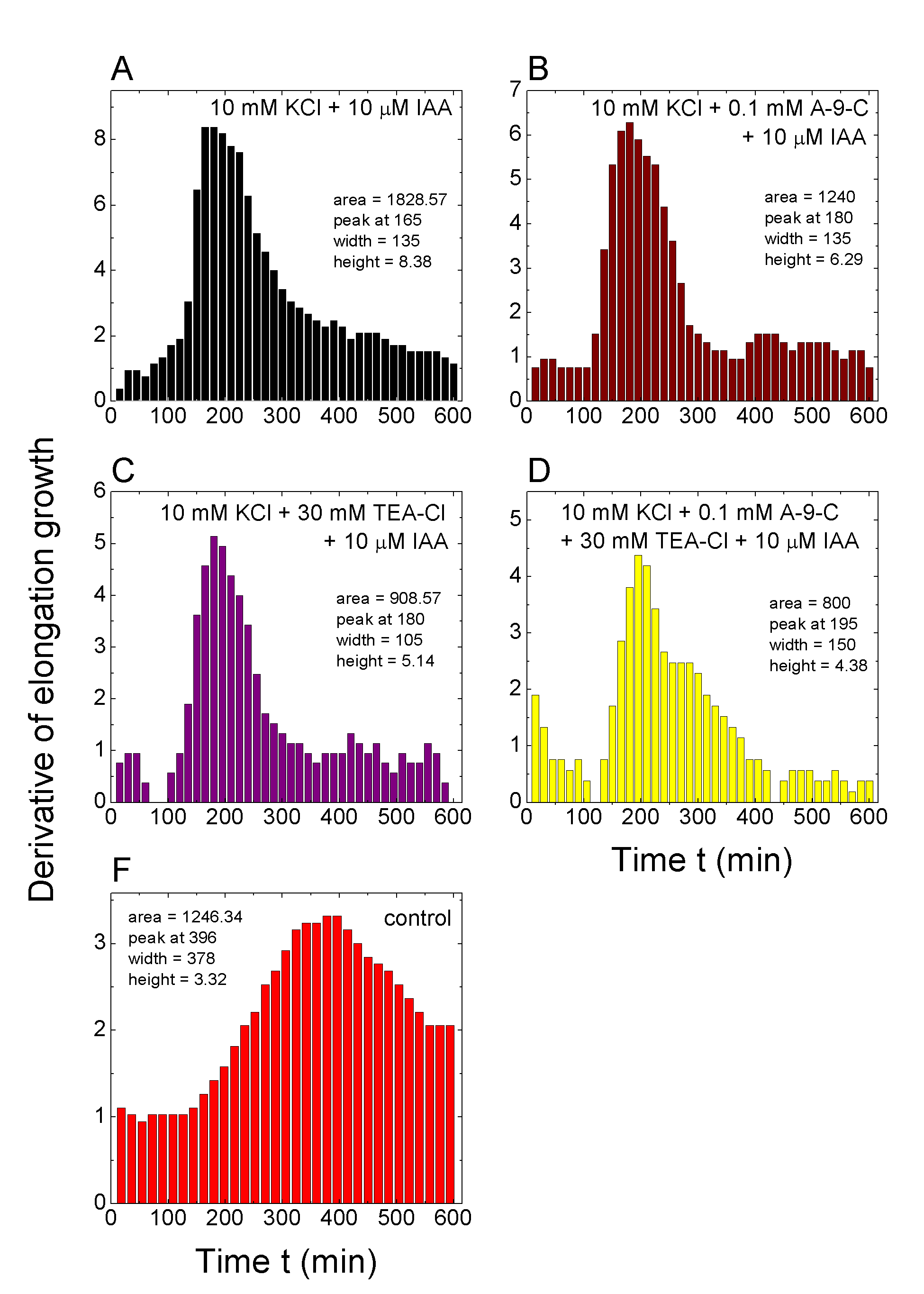
**

**SI Figure 18**

**
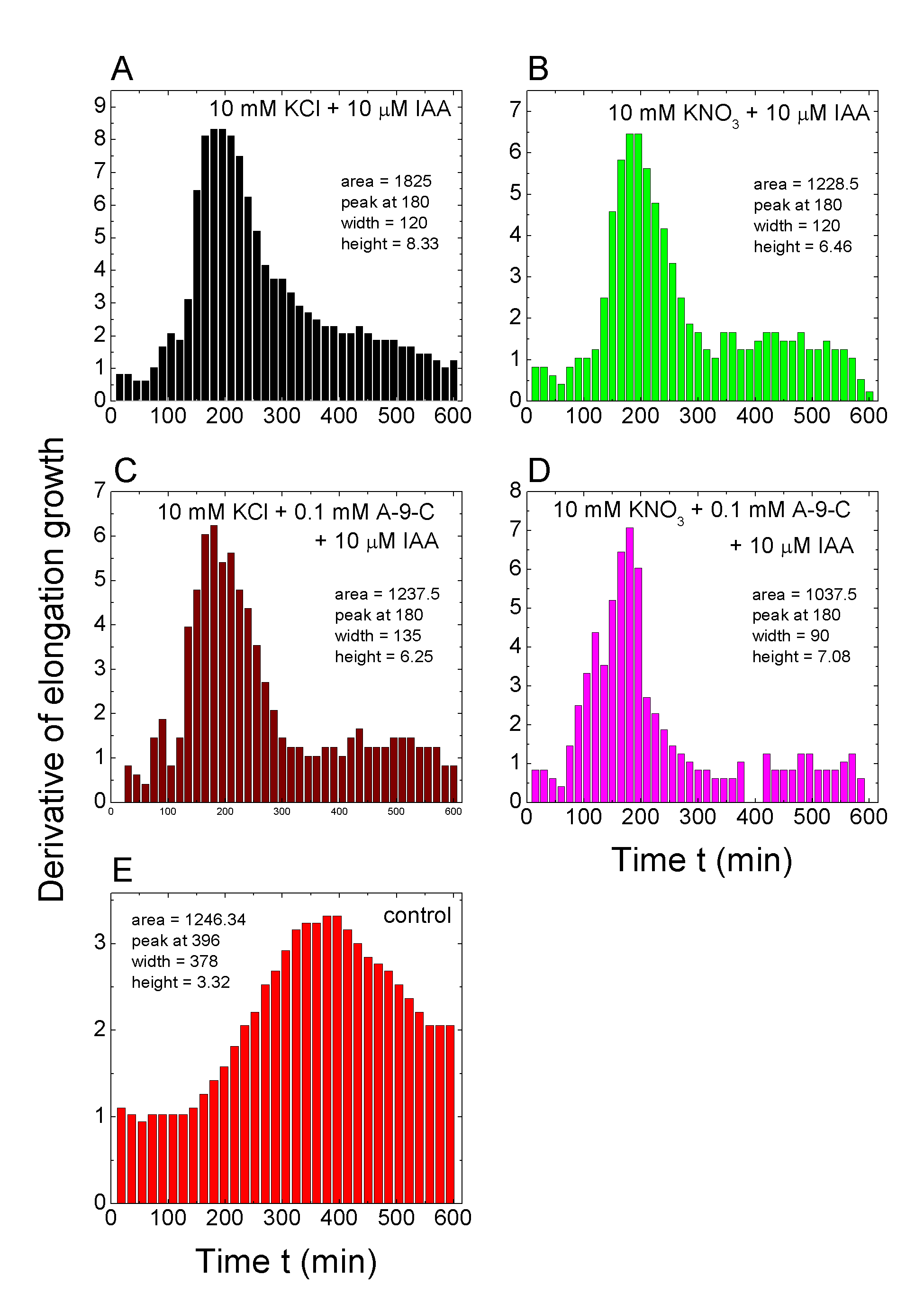
**

**SI Figure 19**
